# Supplementary material for: Trends in gender and socioeconomic inequalities in mental health following the Great Recession and subsequent austerity policies: a repeat cross-sectional analysis of the Health Surveys for England
Source: BMJ Open. 2018 Aug 30;8(8):e022924. doi: 10.1136/bmjopen-2018-022924 (PMC6119415; doi:10.1136/bmjopen-2018-022924)
Supplement: Supplementary file 1 [file bmjopen-2018-022924supp001.pdf]

**Supplementary Appendix**

**Appendix 1: Characteristics of study participants**

| | | Gender (%) | Age Group (%) | | | | Highest Education Level (%) | | | | | |
|---|---|---|---|---|---|---|---|---|---|---|---|---|
| **Year** | | | **25-34** | **35-44** | **45-54** | **55-64** | **Degree** | **A-Level** | **GCSE** | **None** | **Sample** | **Total Sample** |
| **1991** | Male | 46.6 | 29.7 | 27.6 | 21.9 | 20.8 | 16.0 | 20.1 | 32.1 | 31.9 | 942 | |
| | Female | 53.4 | 30.0 | 27.2 | 21.7 | 21.2 | 8.1 | 17.3 | 34.0 | 40.6 | 1078 | **2020** |
| **1992** | Male | 48.4 | 28.6 | 28.4 | 23.8 | 19.2 | 15.2 | 26.0 | 32.5 | 26.3 | 1184 | |
| | Female | 51.6 | 29.8 | 28.4 | 21.7 | 20.1 | 8.7 | 18.5 | 37.9 | 34.9 | 1316 | **2500** |
| **1993** | Male | 47.6 | 29.2 | 26.1 | 24.9 | 19.8 | 16.4 | 25.4 | 30.1 | 28.2 | 5030 | |
| | Female | 52.4 | 30.2 | 27.0 | 23.8 | 19.0 | 9.6 | 18.2 | 35.9 | 36.2 | 5544 | **10,574** |
| **1994** | Male | 46.7 | 29.5 | 27.3 | 23.0 | 20.2 | 15.9 | 26.5 | 30.0 | 27.5 | 4704 | |
| | Female | 53.3 | 31.0 | 27.2 | 23.1 | 18.8 | 10.4 | 18.0 | 37.9 | 33.7 | 5361 | **10,065** |
| **1997** | Male | 47.2 | 27.7 | 27.4 | 25.5 | 19.4 | 19.2 | 28.7 | 28.3 | 23.8 | 2559 | |
| | Female | 52.8 | 30.6 | 27.2 | 24.5 | 17.7 | 14.0 | 21.3 | 33.7 | 31.0 | 2860 | **5419** |
| **1998** | Male | 46.6 | 27.7 | 26.9 | 25.8 | 19.6 | 19.5 | 28.0 | 29.5 | 23.1 | 4582 | |
| | Female | 53.4 | 29.0 | 28.0 | 24.7 | 18.3 | 14.3 | 20.6 | 36.0 | 29.2 | 5254 | **9836** |
| **1999** | Male | 47.0 | 25.4 | 28.1 | 25.4 | 21.0 | 21.0 | 28.5 | 27.5 | 23.0 | 2257 | |
| | Female | 53.0 | 27.4 | 29.9 | 25.7 | 17.0 | 15.5 | 21.6 | 34.4 | 28.6 | 2543 | **4800** |
| **2000** | Male | 45.8 | 25.9 | 29.7 | 23.4 | 21.0 | 21.7 | 30.3 | 27.0 | 21.0 | 2311 | |
| | Female | 54.2 | 27.6 | 30.5 | 23.3 | 18.5 | 16.5 | 25.0 | 34.1 | 24.4 | 2733 | **5044** |
| **2001** | Male | 45.6 | 24.2 | 28.3 | 25.6 | 21.9 | 22.7 | 29.2 | 29.4 | 18.7 | 4360 | |
| | Female | 54.4 | 25.6 | 30.7 | 25.0 | 18.7 | 17.2 | 23.6 | 35.3 | 23.9 | 5193 | **9553** |
| **2002** | Male | 43.4 | 23.0 | 31.5 | 23.8 | 21.7 | 23.1 | 31.6 | 28.2 | 17.2 | 2016 | |
| | Female | 56.6 | 27.8 | 32.1 | 22.0 | 18.2 | 19.4 | 25.1 | 36.2 | 19.3 | 2633 | **4649** |
| **2003** | Male | 45.5 | 22.6 | 28.6 | 24.4 | 24.3 | 23.6 | 28.0 | 29.7 | 18.7 | 4117 | |
| | Female | 54.6 | 23.9 | 30.6 | 23.3 | 22.1 | 20.4 | 24.0 | 34.8 | 20.8 | 4941 | **9058** |

| | | | | | | | | | | | |
|---|---|---|---|---|---|---|---|---|---|---|---|
| **2004** | Male | 43.4 | 23.2 | 27.7 | 22.8 | 26.3 | 25.4 | 27.7 | 27.1 | 19.9 | 1782 | |
| | Female | 56.6 | 21.8 | 30.3 | 24.3 | 23.5 | 21.1 | 24.3 | 31.9 | 22.8 | 2327 | **4109** |
| **2005** | Male | 44.8 | 22.0 | 25.0 | 26.9 | 26.0 | 25.5 | 29.0 | 26.7 | 18.8 | 2076 | |
| | Female | 55.2 | 22.9 | 27.9 | 26.1 | 23.2 | 22.1 | 23.4 | 32.6 | 21.9 | 2555 | **4631** |
| **2006** | Male | 44.8 | 19.9 | 28.2 | 25.0 | 26.9 | 27.2 | 29.9 | 25.6 | 17.3 | 3896 | |
| | Female | 55.2 | 22.2 | 29.2 | 24.7 | 23.8 | 24.2 | 24.3 | 32.1 | 19.4 | 4795 | **8691** |
| **2008** | Male | 44.8 | 21.1 | 27.2 | 24.8 | 26.9 | 27.2 | 30.1 | 25.9 | 16.8 | 4165 | |
| | Female | 55.2 | 22.7 | 27.9 | 25.4 | 24.1 | 24.5 | 26.3 | 30.9 | 18.3 | 5135 | **9300** |
| **2009** | Male | 45.6 | 20.7 | 27.8 | 25.9 | 25.7 | 27.4 | 26.8 | 29.3 | 16.5 | 1278 | |
| | Female | 54.5 | 22.6 | 29.7 | 24.2 | 23.4 | 25.9 | 24.4 | 32.7 | 17.2 | 1528 | **2806** |
| **2010** | Male | 43.5 | 20.3 | 26.1 | 26.7 | 26.8 | 29.6 | 30.2 | 26.6 | 13.6 | 2123 | |
| | Female | 56.5 | 22.1 | 26.8 | 28.2 | 22.9 | 27.5 | 26.6 | 32.0 | 13.9 | 2759 | **4882** |
| **2012** | Male | 43.6 | 19.8 | 26.1 | 26.8 | 27.4 | 32.2 | 29.8 | 24.3 | 13.7 | 2025 | |
| | Female | 56.5 | 22.9 | 26.1 | 28.2 | 22.9 | 32.3 | 27.1 | 26.5 | 14.1 | 2625 | **4650** |
| **2014** | Male | 43.3 | 19.2 | 26.7 | 29.8 | 24.3 | 34.2 | 27.9 | 24.6 | 13.4 | 1964 | |
| | Female | 56.7 | 22.7 | 27.6 | 28.3 | 21.4 | 33.6 | 28.3 | 25.8 | 12.4 | 2568 | **4532** |

| | | **Gender (%)** | **Age Group (%)** | | | | **IMD Quintile** | | | | | | |
|---|---|---|---|---|---|---|---|---|---|---|---|---|---|
| **Year** | | | **25-34** | **35-44** | **45-54** | **55-64** | **1 (Least Deprived)** | **2** | **3** | **4** | **5 (Most Deprived)** | **Sample** | **Total Sample** |
| **2001** | Male | 44.8 | 24.0 | 27.8 | 26.1 | 22.2 | 16.7 | 15.2 | 19.2 | 21.0 | 28.0 | 4211 | |
| | Female | 55.2 | 24.8 | 29.6 | 25.6 | 20.1 | 16.4 | 14.8 | 20.0 | 22.1 | 26.8 | 5184 | **9395** |
| **2002** | Male | 42.7 | 22.9 | 31.1 | 23.9 | 22.1 | 16.6 | 16.6 | 18.6 | 22.2 | 26.0 | 2080 | |
| | Female | 57.3 | 26.8 | 31.0 | 22.7 | 19.6 | 17.2 | 16.0 | 19.0 | 21.6 | 26.2 | 2794 | **4874** |
| **2003** | Male | 44.6 | 22.7 | 28.4 | 24.5 | 24.5 | 22.6 | 20.8 | 19.9 | 20.7 | 16.1 | 4204 | |
| | Female | 55.4 | 23.1 | 29.7 | 23.4 | 23.8 | 23.2 | 20.1 | 19.8 | 20.7 | 16.2 | 5229 | **9433** |
| **2004** | Male | 42.9 | 23.2 | 27.7 | 22.8 | 26.3 | 23.4 | 23.2 | 19.1 | 20.3 | 14.1 | 1786 | |
| | Female | 57.1 | 21.3 | 29.8 | 24.3 | 24.6 | 22.3 | 22.8 | 17.8 | 19.9 | 17.2 | 2380 | **4166** |

| | | | | | | | | | | | | |
|---|---|---|---|---|---|---|---|---|---|---|---|---|
| **2005** | Male | 44.3 | 22.0 | 25.0 | 26.9 | 26.2 | 23.2 | 21.3 | 20.5 | 20.6 | 14.5 | 2082 | |
| | Female | 55.7 | 22.4 | 27.4 | 25.9 | 24.3 | 22.2 | 21.6 | 19.1 | 20.7 | 16.4 | 2618 | **4700** |
| **2006** | Male | 44.4 | 19.8 | 28.2 | 25.0 | 26.9 | 20.9 | 21.4 | 21.4 | 19.8 | 16.5 | 3907 | |
| | Female | 55.6 | 22.0 | 28.8 | 24.6 | 24.7 | 20.2 | 22.7 | 21.0 | 19.4 | 16.7 | 4888 | **8795** |
| **2008** | Male | 44.4 | 21.1 | 27.2 | 24.7 | 27.0 | 22.3 | 20.3 | 19.9 | 19.4 | 18.1 | 4169 | |
| | Female | 55.6 | 22.3 | 27.5 | 25.2 | 25.0 | 22.6 | 19.9 | 20.0 | 19.2 | 18.3 | 5230 | **9399** |
| **2009** | Male | 45.1 | 20.6 | 27.8 | 25.8 | 25.8 | 20.4 | 22.4 | 20.4 | 20.0 | 16.9 | 1282 | |
| | Female | 54.9 | 22.3 | 29.3 | 24.3 | 24.2 | 20.6 | 21.4 | 21.2 | 20.0 | 16.7 | 1559 | **2841** |
| **2010** | Male | 43.1 | 20.3 | 26.1 | 26.7 | 26.9 | 23.4 | 19.8 | 20.0 | 19.0 | 17.9 | 2129 | |
| | Female | 56.9 | 21.9 | 26.6 | 28.1 | 23.6 | 23.7 | 19.7 | 20.0 | 19.5 | 17.3 | 2806 | **4935** |
| **2012** | Male | 43.4 | 19.8 | 26.1 | 26.9 | 27.3 | 22.1 | 21.1 | 20.2 | 19.3 | 17.4 | 2030 | |
| | Female | 56.6 | 22.8 | 25.9 | 28.0 | 23.4 | 22.7 | 20.7 | 19.9 | 19.2 | 17.6 | 2649 | **4679** |
| **2014** | Male | 43.2 | 19.3 | 26.6 | 29.9 | 24.3 | 23.5 | 20.3 | 18.4 | 18.7 | 19.1 | 1969 | |
| | Female | 56.8 | 22.6 | 27.6 | 28.1 | 21.8 | 21.7 | 19.6 | 20.0 | 19.7 | 19.1 | 2592 | **4561** |

**Appendix 2: Age-standardised GHQ caseness in men and women aged 25-64 years, 1991-2014**

(displayed in Figure 1 in main text)

| | Men | | | | Women | | | |
|---|---|---|---|---|---|---|---|---|
| Year | Sample Size | Adjusted Est. (%) | Lower 95% CI | Upper 95% CI | Sample Size | Adjusted Est. (%) | Lower 95% CI | Upper 95% CI |
| **1991** | 942 | 12.17 | 10.06 | 14.28 | 1078 | 19.46 | 17.08 | 21.84 |
| **1992** | 1184 | 14.48 | 12.44 | 16.52 | 1316 | 19.71 | 17.52 | 21.90 |
| **1993** | 5030 | 14.04 | 13.07 | 15.01 | 5544 | 18.82 | 17.78 | 19.86 |
| **1994** | 4704 | 13.04 | 12.06 | 14.02 | 5361 | 19.17 | 18.10 | 20.24 |
| **1997** | 2559 | 12.77 | 11.47 | 14.07 | 2860 | 19.06 | 17.60 | 20.52 |
| **1998** | 4582 | 13.14 | 12.15 | 14.13 | 5254 | 17.98 | 16.94 | 19.03 |
| **1999** | 2257 | 15.32 | 13.83 | 16.81 | 2543 | 18.87 | 17.33 | 20.41 |
| **2000** | 2311 | 11.97 | 10.63 | 13.30 | 2733 | 16.44 | 15.03 | 17.85 |
| **2001** | 4360 | 11.38 | 10.43 | 12.32 | 5193 | 15.30 | 14.31 | 16.28 |
| **2002** | 2016 | 14.35 | 12.81 | 15.89 | 2633 | 18.97 | 17.44 | 20.50 |
| **2003** | 4117 | 11.86 | 10.87 | 12.85 | 4941 | 14.80 | 13.81 | 15.80 |
| **2004** | 1782 | 11.66 | 10.16 | 13.17 | 2327 | 15.18 | 13.71 | 16.64 |
| **2005** | 2076 | 11.57 | 10.19 | 12.94 | 2555 | 15.99 | 14.57 | 17.41 |
| **2006** | 3896 | 11.95 | 10.93 | 12.98 | 4795 | 15.58 | 14.55 | 16.61 |
| **2008** | 4165 | 11.62 | 10.64 | 12.60 | 5135 | 16.01 | 15.01 | 17.02 |
| **2009** | 1278 | 16.80 | 14.75 | 18.86 | 1528 | 17.13 | 15.23 | 19.04 |
| **2010** | 2123 | 14.58 | 13.07 | 16.09 | 2759 | 16.66 | 15.27 | 18.05 |
| **2012** | 2025 | 12.67 | 11.22 | 14.11 | 2625 | 18.72 | 17.22 | 20.21 |
| **2014** | 1964 | 12.90 | 11.40 | 14.40 | 2568 | 17.67 | 16.19 | 19.15 |

**Appendix 3: Adjusted odds ratio and % point difference in GHQ caseness by gender, 1991-2014**

(displayed in Figure 1 in main text)

| | Model 1: Adjusted for age | | | Model 2: Adjusted for age, education, employment | | |
|---|---|---|---|---|---|---|
| **Year** | **OR (95% CI)** | **p-value** | **% Diff. (95% CI)** | **OR (95% CI)** | **p-value** | **% Diff. (95% CI)** |
| **1991** | 1.76 (1.38-2.26) | <0.001 | 7.61 (4.31-10.90) | 1.83 (1.39-2.40) | <0.001 | 7.85 (4.33-11.37) |
| **1992** | 1.48 (1.20-1.83) | <0.001 | 5.53 (2.53-8.52) | 1.57 (1.24-1.98) | <0.001 | 6.23 (2.95-9.50) |
| **1993** | 1.43 (1.28-1.58) | <0.001 | 4.91 (3.47-6.35) | 1.52 (1.35-1.70) | <0.001 | 5.64 (4.11-7.17) |
| **1994** | 1.60 (1.44-1.79) | <0.001 | 6.42 (4.94-7.89) | 1.76 (1.57-1.98) | <0.001 | 7.44 (5.89-9.00) |
| **1997** | 1.60 (1.38-1.85) | <0.001 | 6.31 (4.31-8.31) | 1.68 (1.43-1.98) | <0.001 | 6.73 (4.63-8.84) |
| **1998** | 1.47 (1.32-1.64) | <0.001 | 5.11 (3.65-6.58) | 1.58 (1.40-1.78) | <0.001 | 5.79 (4.26-7.33) |
| **1999** | 1.29 (1.11-1.51) | 0.001 | 3.68 (1.52-5.83) | 1.41 (1.20-1.67) | <0.001 | 4.67 (2.42-6.91) |
| **2000** | 1.45 (1.23-1.70) | <0.001 | 4.56 (2.58-6.54) | 1.62 (1.35-1.95) | <0.001 | 5.52 (3.43-7.61) |
| **2001** | 1.41 (1.25-1.59) | <0.001 | 4.04 (2.64-5.45) | 1.51 (1.32-1.72) | <0.001 | 4.55 (3.10-6.01) |
| **2002** | 1.41 (1.20-1.65) | <0.001 | 4.77 (2.55-6.98) | 1.50 (1.26-1.79) | <0.001 | 5.41 (3.11-7.71) |
| **2003** | 1.27 (1.12-1.44) | <0.001 | 2.74 (1.30-4.17) | 1.34 (1.17-1.54) | <0.001 | 3.19 (1.69-4.69) |
| **2004** | 1.33 (1.10-1.60) | 0.004 | 3.22 (1.07-5.37) | 1.36 (1.10-1.69) | 0.004 | 3.32 (1.06-5.57) |
| **2005** | 1.49 (1.25-1.78) | <0.001 | 4.68 (2.67-6.70) | 1.65 (1.36-2.01) | <0.001 | 5.39 (3.32-7.45) |
| **2006** | 1.32 (1.16-1.50) | <0.001 | 3.23 (1.73-4.72) | 1.39 (1.21-1.60) | <0.001 | 3.54 (2.03-5.05) |
| **2008** | 1.45 (1.28-1.64) | <0.001 | 4.35 (2.91-5.79) | 1.53 (1.33-1.75) | <0.001 | 4.53 (3.07-5.99) |
| **2009** | 0.98 (0.80-1.21) | 0.866 | -0.25 (-3.12-2.62) | 1.09 (0.87-1.37) | 0.446 | 1.12 (-1.75-3.98) |
| **2010** | 1.17 (0.99-1.39) | 0.065 | 2.08 (-0.11-4.27) | 1.22 (1.01-1.46) | 0.034 | 2.39 (0.19-4.58) |
| **2012** | 1.57 (1.32-1.86) | <0.001 | 5.93 (3.70-8.15) | 1.79 (1.48-2.16) | <0.001 | 6.78 (4.61-8.94) |
| **2014** | 1.42 (1.19-1.70) | <0.001 | 4.53 (2.28-6.78) | 1.43 (1.19-1.71) | <0.001 | 4.51 (2.27-6.75) |

**Appendix 4: Full multiple logistic regression models for participants of each gender, 2001-2014**

| Regression models for men (n=24,930) | | | | | | | | | | | | |
|---|---|---|---|---|---|---|---|---|---|---|---|---|
| | Model 1: Adjusted for age, IMD | | | | Model 2: Adjusted for age, IMD, employment | | | | Model 3: Adjusted for age, IMD, employment, income | | | |
| **Year** | **Odds Ratio** | **p-value** | **Lower 95% CI** | **Upper 95% CI** | **Odds Ratio** | **p-value** | **Lower 95% CI** | **Upper 95% CI** | **Odds Ratio** | **p-value** | **Lower 95% CI** | **Upper 95% CI** |
| **2001** | 0.93 | 0.330 | 0.80 | 1.08 | 0.89 | 0.147 | 0.76 | 1.04 | 0.86 | 0.069 | 0.73 | 1.01 |
| **2002** | 1.27 | 0.007 | 1.07 | 1.51 | 1.29 | 0.006 | 1.07 | 1.54 | 1.26 | 0.014 | 1.05 | 1.51 |
| **2003** | 1.06 | 0.481 | 0.91 | 1.23 | 0.97 | 0.667 | 0.82 | 1.13 | 0.94 | 0.475 | 0.80 | 1.11 |
| **2004** | 1.11 | 0.272 | 0.92 | 1.35 | 1.07 | 0.505 | 0.87 | 1.31 | 1.05 | 0.646 | 0.86 | 1.29 |
| **2005** | 1.03 | 0.722 | 0.86 | 1.24 | 0.92 | 0.400 | 0.76 | 1.12 | 0.90 | 0.303 | 0.74 | 1.10 |
| **2006** | 1.06 | 0.476 | 0.91 | 1.24 | 1.04 | 0.676 | 0.88 | 1.22 | 1.03 | 0.765 | 0.87 | 1.21 |
| **2008** | 1.00 | - | - | - | 1.00 | - | - | - | 1.00 | - | - | - |
| **2009** | 1.64 | <0.001 | 1.34 | 2.00 | 1.55 | <0.001 | 1.25 | 1.93 | 1.53 | <0.001 | 1.24 | 1.91 |
| **2010** | 1.28 | 0.009 | 1.06 | 1.53 | 1.26 | 0.021 | 1.04 | 1.52 | 1.26 | 0.018 | 1.04 | 1.53 |
| **2012** | 1.15 | 0.147 | 0.95 | 1.38 | 1.10 | 0.340 | 0.91 | 1.33 | 1.10 | 0.342 | 0.91 | 1.33 |
| **2014** | 1.13 | 0.215 | 0.93 | 1.37 | 1.17 | 0.126 | 0.96 | 1.43 | 1.18 | 0.108 | 0.96 | 1.44 |

| Regression models for women (n=31,413) | | | | | | | | | | | | |
|---|---|---|---|---|---|---|---|---|---|---|---|---|
| | Model 1: Adjusted for age, IMD | | | | Model 2: Adjusted for age, IMD, employment | | | | Model 3: Adjusted for age, IMD, employment, income | | | |
| **Year** | **Odds Ratio** | **p-value** | **Lower 95% CI** | **Upper 95% CI** | **Odds Ratio** | **p-value** | **Lower 95% CI** | **Upper 95% CI** | **Odds Ratio** | **p-value** | **Lower 95% CI** | **Upper 95% CI** |
| **2001** | 0.94 | 0.280 | 0.83 | 1.05 | 0.91 | 0.143 | 0.81 | 1.03 | 0.87 | 0.030 | 0.78 | 0.99 |
| **2002** | 1.14 | 0.049 | 1.00 | 1.31 | 1.13 | 0.078 | 0.99 | 1.30 | 1.10 | 0.162 | 0.96 | 1.27 |
| **2003** | 0.92 | 0.192 | 0.82 | 1.04 | 0.91 | 0.112 | 0.80 | 1.02 | 0.87 | 0.026 | 0.77 | 0.98 |
| **2004** | 0.93 | 0.358 | 0.80 | 1.08 | 0.91 | 0.218 | 0.78 | 1.06 | 0.88 | 0.112 | 0.75 | 1.03 |
| **2005** | 1.06 | 0.426 | 0.92 | 1.22 | 1.05 | 0.531 | 0.91 | 1.21 | 1.02 | 0.762 | 0.88 | 1.18 |
| **2006** | 0.91 | 0.153 | 0.81 | 1.03 | 0.90 | 0.102 | 0.80 | 1.02 | 0.89 | 0.077 | 0.79 | 1.01 |
| **2008** | 1.00 | - | - | - | 1.00 | - | - | - | 1.00 | - | - | - |
| **2009** | 1.06 | 0.537 | 0.89 | 1.25 | 1.07 | 0.429 | 0.90 | 1.28 | 1.07 | 0.431 | 0.90 | 1.28 |
| **2010** | 1.00 | 0.980 | 0.86 | 1.15 | 0.99 | 0.849 | 0.85 | 1.14 | 0.99 | 0.922 | 0.86 | 1.15 |
| **2012** | 1.24 | 0.003 | 1.08 | 1.42 | 1.24 | 0.003 | 1.07 | 1.43 | 1.25 | 0.002 | 1.09 | 1.45 |
| **2014** | 1.10 | 0.208 | 0.95 | 1.27 | 1.11 | 0.171 | 0.96 | 1.28 | 1.12 | 0.138 | 0.97 | 1.29 |

**Appendix 5: Age-sex standardised GHQ caseness by IMD quintile in 25–64-year-olds, 2001-2014** (displayed in Figure 2 in main text)

| Year | Quintile | Sample Size | Adjusted Est. (%) | Lower 95% CI | Upper 95% CI | Year | Quintile | Sample Size | Adjusted Est. (%) | Lower 95% CI | Upper 95% CI |
|---|---|---|---|---|---|---|---|---|---|---|---|
| **2001** | Least Dep. | 1549 | 11.80 | 10.20 | 13.41 | **2002** | Least Dep. | 825 | 12.65 | 10.35 | 14.94 |
| | 2 | 1403 | 9.36 | 7.83 | 10.88 | | 2 | 794 | 13.74 | 11.37 | 16.11 |
| | 3 | 1846 | 13.38 | 11.83 | 14.94 | | 3 | 916 | 13.26 | 11.11 | 15.42 |
| | 4 | 2030 | 14.06 | 12.56 | 15.57 | | 4 | 1065 | 19.99 | 17.52 | 22.46 |
| | Most Dep. | 2567 | 16.42 | 14.97 | 17.88 | | Most Dep. | 1274 | 20.94 | 18.64 | 23.24 |
| **2003** | Least Dep. | 2163 | 11.38 | 10.00 | 12.75 | **2004** | Least Dep. | 948 | 11.98 | 9.73 | 14.22 |
| | 2 | 1924 | 11.19 | 9.78 | 12.61 | | 2 | 957 | 10.79 | 8.78 | 12.80 |
| | 3 | 1872 | 10.98 | 9.55 | 12.40 | | 3 | 764 | 11.07 | 8.87 | 13.28 |
| | 4 | 1950 | 16.18 | 14.54 | 17.83 | | 4 | 836 | 15.66 | 13.18 | 18.13 |
| | Most Dep. | 1524 | 18.51 | 16.53 | 20.49 | | Most Dep. | 661 | 19.56 | 16.43 | 22.70 |
| **2005** | Least Dep. | 1064 | 9.55 | 7.73 | 11.37 | **2006** | Least Dep. | 1806 | 10.27 | 8.81 | 11.73 |
| | 2 | 1008 | 12.23 | 10.22 | 14.24 | | 2 | 1943 | 12.50 | 11.00 | 13.99 |
| | 3 | 927 | 12.18 | 10.08 | 14.27 | | 3 | 1864 | 11.75 | 10.28 | 13.21 |
| | 4 | 970 | 15.05 | 12.81 | 17.28 | | 4 | 1719 | 15.71 | 13.97 | 17.45 |
| | Most Dep. | 731 | 23.72 | 20.53 | 26.92 | | Most Dep. | 1463 | 20.68 | 18.56 | 22.80 |
| **2008** | Least Dep. | 2108 | 10.44 | 9.10 | 11.78 | **2009** | Least Dep. | 582 | 14.92 | 11.95 | 17.90 |
| | 2 | 1889 | 11.11 | 9.67 | 12.54 | | 2 | 621 | 11.09 | 8.62 | 13.57 |
| | 3 | 1877 | 12.83 | 11.33 | 14.33 | | 3 | 593 | 17.91 | 14.79 | 21.03 |
| | 4 | 1815 | 15.12 | 13.47 | 16.76 | | 4 | 568 | 19.51 | 16.21 | 22.81 |
| | Most Dep. | 1710 | 20.54 | 18.59 | 22.50 | | Most Dep. | 477 | 24.07 | 20.06 | 28.08 |
| **2010** | Least Dep. | 1162 | 13.73 | 11.66 | 15.81 | **2012** | Least Dep. | 1048 | 10.67 | 8.72 | 12.62 |
| | 2 | 973 | 11.60 | 9.55 | 13.65 | | 2 | 977 | 14.40 | 12.22 | 16.58 |
| | 3 | 985 | 15.53 | 13.28 | 17.78 | | 3 | 937 | 14.50 | 12.26 | 16.75 |
| | 4 | 951 | 17.48 | 15.05 | 19.92 | | 4 | 899 | 16.83 | 14.40 | 19.26 |
| | Most Dep. | 864 | 22.28 | 19.47 | 25.09 | | Most Dep. | 818 | 24.18 | 21.21 | 27.16 |
| **2014** | Least Dep. | 1024 | 10.71 | 8.80 | 12.62 | | | | | | |
| | 2 | 907 | 13.52 | 11.27 | 15.76 | | | | | | |
| | 3 | 880 | 15.30 | 12.93 | 17.67 | | | | | | |
| | 4 | 879 | 16.04 | 13.61 | 18.48 | | | | | | |
| | Most Dep. | 871 | 21.91 | 19.10 | 24.73 | | | | | | |

**Appendix 6: Age-sex standardised GHQ caseness by education level in 25–64-year-olds, 1991-2014**

(displayed in Figure 3 in main text)

| Year | Educ. Level | Sample Size | Adjusted Est. (%) | Lower 95% CI | Upper 95% CI | Year | Educ. Level | Sample Size | Adjusted Est. (%) | Lower 95% CI | Upper 95% CI |
|---|---|---|---|---|---|---|---|---|---|---|---|
| **1991** | Degree | 238 | 16.92 | 11.61 | 22.24 | **1992** | Degree | 295 | 16.88 | 12.67 | 21.09 |
| | A-Level | 375 | 19.29 | 14.49 | 24.08 | | A-Level | 551 | 16.69 | 13.28 | 20.10 |
| | GCSE | 669 | 13.85 | 11.15 | 16.54 | | GCSE | 884 | 17.08 | 14.36 | 19.79 |
| | None | 738 | 15.65 | 12.81 | 18.48 | | None | 770 | 16.57 | 13.83 | 19.30 |
| **1993** | Degree | 1356 | 18.18 | 15.56 | 20.79 | **1994** | Degree | 1308 | 17.61 | 15.39 | 19.82 |
| | A-Level | 2286 | 15.55 | 13.96 | 17.14 | | A-Level | 2213 | 15.20 | 13.57 | 16.83 |
| | GCSE | 3508 | 15.90 | 14.61 | 17.18 | | GCSE | 3445 | 14.83 | 13.55 | 16.11 |
| | None | 3424 | 17.34 | 15.98 | 18.70 | | None | 3099 | 17.03 | 15.65 | 18.41 |
| **1997** | Degree | 892 | 12.23 | 9.97 | 14.49 | **1998** | Degree | 1642 | 14.66 | 12.75 | 16.56 |
| | A-Level | 1344 | 16.23 | 14.13 | 18.32 | | A-Level | 2362 | 15.38 | 13.88 | 16.88 |
| | GCSE | 1687 | 15.01 | 13.21 | 16.80 | | GCSE | 3243 | 15.23 | 13.90 | 16.56 |
| | None | 1496 | 16.69 | 14.69 | 18.70 | | None | 2589 | 16.92 | 15.30 | 18.53 |
| **1999** | Degree | 868 | 15.62 | 13.07 | 18.17 | **2000** | Degree | 952 | 12.50 | 10.31 | 14.68 |
| | A-Level | 1193 | 15.32 | 13.20 | 17.45 | | A-Level | 1382 | 10.75 | 9.08 | 12.42 |
| | GCSE | 1494 | 16.36 | 14.37 | 18.36 | | GCSE | 1558 | 14.38 | 12.53 | 16.23 |
| | None | 1245 | 21.39 | 18.80 | 23.98 | | None | 1152 | 19.12 | 16.45 | 21.80 |
| **2001** | Degree | 1881 | 12.40 | 10.82 | 13.99 | **2002** | Degree | 975 | 14.01 | 11.80 | 16.23 |
| | A-Level | 2501 | 12.87 | 11.54 | 14.20 | | A-Level | 1299 | 15.52 | 13.45 | 17.59 |
| | GCSE | 3115 | 12.47 | 11.27 | 13.67 | | GCSE | 1520 | 16.46 | 14.46 | 18.46 |
| | None | 2056 | 15.93 | 14.08 | 17.78 | | None | 855 | 22.12 | 18.83 | 25.42 |
| **2003** | Degree | 1982 | 11.25 | 9.81 | 12.68 | **2004** | Degree | 943 | 13.10 | 10.86 | 15.35 |
| | A-Level | 2339 | 12.22 | 10.87 | 13.57 | | A-Level | 1058 | 12.41 | 10.44 | 14.39 |
| | GCSE | 2940 | 12.98 | 11.74 | 14.22 | | GCSE | 1224 | 10.94 | 9.15 | 12.73 |
| | None | 1797 | 17.16 | 15.05 | 19.27 | | None | 884 | 18.26 | 15.36 | 21.17 |
| **2005** | Degree | 1094 | 10.98 | 9.08 | 12.87 | **2006** | Degree | 2217 | 10.51 | 9.19 | 11.83 |
| | A-Level | 1199 | 12.65 | 10.76 | 14.53 | | A-Level | 2332 | 13.37 | 11.98 | 14.76 |
| | GCSE | 1389 | 13.56 | 11.75 | 15.37 | | GCSE | 2536 | 13.73 | 12.36 | 15.11 |
| | None | 949 | 19.51 | 16.52 | 22.50 | | None | 1606 | 19.69 | 17.47 | 21.91 |
| **2008** | Degree | 2388 | 11.04 | 9.76 | 12.32 | **2009** | Degree | 745 | 13.01 | 10.43 | 15.59 |
| | A-Level | 2604 | 12.55 | 11.28 | 13.83 | | A-Level | 715 | 16.15 | 13.42 | 18.87 |
| | GCSE | 2669 | 14.82 | 13.44 | 16.20 | | GCSE | 873 | 18.41 | 15.76 | 21.06 |
| | None | 1639 | 19.43 | 17.18 | 21.68 | | None | 473 | 21.60 | 17.46 | 25.74 |
| **2010** | Degree | 1387 | 13.21 | 11.40 | 15.02 | **2012** | Degree | 1500 | 14.63 | 12.85 | 16.41 |
| | A-Level | 1375 | 15.43 | 13.51 | 17.35 | | A-Level | 1314 | 14.17 | 12.30 | 16.05 |
| | GCSE | 1447 | 15.56 | 13.64 | 17.49 | | GCSE | 1188 | 16.87 | 14.69 | 19.06 |
| | None | 673 | 21.54 | 17.86 | 25.22 | | None | 648 | 19.35 | 16.07 | 22.63 |
| **2014** | Degree | 1533 | 12.96 | 11.25 | 14.67 | | | | | | |
| | A-Level | 1273 | 13.61 | 11.68 | 15.53 | | | | | | |

| | | | | | | | | | | | |
|---|---|---|---|---|---|---|---|---|---|---|---|
| | GCSE | 1145 | 15.13 | 13.00 | 17.26 | | | | | | |
| | None | 581 | 23.71 | 20.04 | 27.38 | | | | | | |

**Appendix 7: Relative index of inequality in GHQ caseness, 1991-2014**

(displayed in Figure 4 in main text)

| Highest Education Level as Measure of SEP | | | | | Area-Level Deprivation as Measure of SEP | | | | |
|---|---|---|---|---|---|---|---|---|---|
| **Year** | **Incidence Rate Ratio** | **p-value** | **Lower 95% CI** | **Upper 95% CI** | **Year** | **Incidence Rate Ratio** | **p-value** | **Lower 95% CI** | **Upper 95% CI** |
| **1991** | 0.86 | 0.445 | 0.58 | 1.27 | | | | | |
| **1992** | 0.88 | 0.458 | 0.63 | 1.23 | | | | | |
| **1993** | 1.02 | 0.797 | 0.87 | 1.21 | | | | | |
| **1994** | 0.96 | 0.634 | 0.81 | 1.14 | | | | | |
| **1997** | 1.25 | 0.054 | 1.00 | 1.57 | | | | | |
| **1998** | 1.11 | 0.232 | 0.93 | 1.32 | | | | | |
| **1999** | 1.46 | 0.002 | 1.15 | 1.86 | | | | | |
| **2000** | 1.79 | <0.001 | 1.37 | 2.33 | | | | | |
| **2001** | 1.26 | 0.018 | 1.04 | 1.53 | **2001** | 1.68 | <0.001 | 1.40 | 2.02 |
| **2002** | 1.51 | 0.001 | 1.18 | 1.93 | **2002** | 1.93 | <0.001 | 1.54 | 2.41 |
| **2003** | 1.57 | <0.001 | 1.27 | 1.92 | **2003** | 1.95 | <0.001 | 1.61 | 2.37 |
| **2004** | 1.35 | 0.066 | 0.98 | 1.86 | **2004** | 2.05 | <0.001 | 1.52 | 2.77 |
| **2005** | 1.70 | <0.001 | 1.29 | 2.24 | **2005** | 2.65 | <0.001 | 2.03 | 3.47 |
| **2006** | 1.98 | <0.001 | 1.61 | 2.43 | **2006** | 2.13 | <0.001 | 1.74 | 2.60 |
| **2008** | 1.79 | <0.001 | 1.47 | 2.18 | **2008** | 2.28 | <0.001 | 1.89 | 2.76 |
| **2009** | 2.13 | <0.001 | 1.52 | 2.99 | **2009** | 2.11 | <0.001 | 1.54 | 2.91 |
| **2010** | 1.59 | 0.001 | 1.22 | 2.08 | **2010** | 1.85 | <0.001 | 1.43 | 2.38 |
| **2012** | 1.46 | 0.005 | 1.12 | 1.90 | **2012** | 2.32 | <0.001 | 1.80 | 2.99 |
| **2014** | 1.72 | <0.001 | 1.31 | 2.26 | **2014** | 2.22 | <0.001 | 1.72 | 2.87 |
