# Supplementary material for: Trends in gender and socioeconomic inequalities in mental health following the Great Recession and subsequent austerity policies: a repeat cross-sectional analysis of the Health Surveys for England
Source: BMJ Open. 2018 Aug 30;8(8):e022924. doi: 10.1136/bmjopen-2018-022924 (PMC6119415; doi:10.1136/bmjopen-2018-022924)
Supplement: Author's manuscript [file bmjopen-2018-022924.draft_revisions.pdf]

BMJ Open is committed to open peer review. As part of this commitment we make the peer review history of every article we publish publicly available.

When an article is published we post the peer reviewers' comments and the authors' responses online. We also post the versions of the paper that were used during peer review. These are the versions that the peer review comments apply to.

The versions of the paper that follow are the versions that were submitted during the peer review process. They are not the versions of record or the final published versions. They should not be cited or distributed as the published version of this manuscript.

BMJ Open is an open access journal and the full, final, typeset and author-corrected version of record of the manuscript is available on our site with no access controls, subscription charges or pay-per-view fees (http://bmjopen.bmj.com).

If you have any questions on BMJ Open's open peer review process please email info.bmjopen@bmj.com

BMJ Open

Trends in gender and socioeconomic inequalities in mental health in England following the Great Recession and subsequent austerity policies

Journal:	*BMJ Open*
Manuscript ID	bmjopen-2018-022924
Article Type:	Research
Date Submitted by the Author:	16-Mar-2018
Complete List of Authors:	Thomson, Rachel; MRC/CSO Social & Public Health Sciences Unit, University of Glasgow; NHS Ayrshire and Arran, Public Health<br>Niedzwiedz, Claire; MRC/CSO Social & Public Health Sciences Unit, University of Glasgow<br>Katikireddi, Srinivasa; MRC/CSO Social & Public Health Sciences Unit, University of Glasgow
Keywords:	MENTAL HEALTH, PUBLIC HEALTH, SOCIAL MEDICINE, EPIDEMIOLOGY

SCHOLARONE™
Manuscripts

For peer review only

Trends in gender and socioeconomic inequalities in mental health in England following the Great Recession and subsequent austerity policies

Rachel M Thomson*[1, 2], Claire L Niedzwiedz[1], Srinivasa Vittal Katikireddi[1]

[1] MRC/CSO Social & Public Health Sciences Unit, University of Glasgow, UK

[2] Public Health Department, NHS Ayrshire & Arran, UK

*Corresponding author

Postal address:

Dr Rachel Thomson
Public Health Dept, Afton House
Ailsa Hospital Campus
Dalmellington Road
Ayr KA6 6AB

Email rachel.thomson8@nhs.net

Phone: 01292 885 858

Word count (excl. title page, abstract, references, figures and tables, declarations): 3015 words

For peer review only

ABSTRACT

Objective: It is known that mental health deteriorated following the 2008 global financial crisis, and that subsequent UK austerity policies post-2010 disproportionately impacted women and those in deprived areas. We aimed to assess whether gender and socioeconomic inequalities in poor mental health have changed since the onset of austerity policies.

Design: Repeat cross-sectional analysis of survey data.

Setting: England.

Participants: Nationally and regionally representative samples of the working age population (25-64 years) from the Health Survey for England (1991-2014).

Outcome Measures: Population-level poor mental health was measured by General Health Questionnaire-12 (GHQ) caseness, stratified by gender and socioeconomic position (area-level deprivation and highest educational attainment).

Results: The prevalence of age-adjusted male GHQ caseness increased by 5.9% (percentage points; 95% CI 3.2%-8.5%, P<0.001) from 2008 to 2009 in the immediate post-recession period, but recovered to pre-recession levels after 2010. In women, there was little change in 2009 or 2010, but an increase of 3.0% (95% CI 1.0%-5.1%, p=0.004) in 2012 compared to 2008 following the onset of austerity. Estimates were largely unchanged after further adjustment for socioeconomic position, employment status, and household income as potential mediators. Relative socioeconomic inequalities in GHQ caseness narrowed from 2008 to 2010 immediately following the recession, with relative index of inequality (RII) falling from 2.28 (95% CI 1.89-2.76, p<0.001) to 1.85 (95% CI 1.43-2.38, p<0.001), but returned to pre-recession levels during austerity.

Conclusions: Gender inequalities in poor mental health narrowed following the Great Recession but widened during austerity, creating the widest gender gap since 1994. Socioeconomic inequalities in poor mental health narrowed immediately post-recession, but this trend may be now reversing. Austerity policies may contribute to widening mental health inequalities.

Keywords: mental health; public health; social medicine; epidemiology

For peer review only

ARTICLE SUMMARY

What is already known on this subject?

- The 2008 recession was associated with worsening population mental health, with an increase in the prevalence of poor mental health in men but not women in the UK.
- In the UK (and many other countries), austerity policies which include substantial welfare and public sector reforms have been implemented since 2010.

What this study adds?

- Since the onset of austerity policies, there has been a widening of gender inequalities in poor mental health due to worsening mental health among women in 2012, while men's mental health has recovered following the Great Recession.
- Socioeconomic inequalities in poor mental health narrowed immediately following the recession but returned to pre-recession highs by 2014, with those without formal qualifications emerging as a high risk group.
- The potential for adverse mental health consequences of austerity policies should be considered by government policymakers.

Strengths and limitations of this study

- Data are from a large nationally and regionally representative survey, and our study considered trends over a long period of time using a validated measure of poor mental health.
- Inequalities in poor mental health were explored by both socioeconomic status (using two measures to demonstrate consistency of trends) and gender, rarely explicitly done in current literature.
- Lack of available data meant it was not possible to categorise individuals according to whether they were subject to specific austerity measures; further research with such data and a clear control group would strengthen arguments for causality.
- The use of cross-sectional rather than longitudinal data mean the ability to derive causal inferences is limited and further longitudinal work may be required.

For peer review only

INTRODUCTION

The health and social repercussions of the 2008 Great Recession are still being felt today.[1, 2] Much existing research has focused on the relationship between the economic downturn, rises in unemployment, and worsening mental health outcomes.[3, 4] Mirroring historical trends, in the aftermath of the recession there was an improvement in all-cause mortality across Europe,[5] paradoxically accompanied by a sharp rise in suicide rates which disproportionately impacted men.[6]

There has been a growing call to interpret trends in mental health outcomes in the context of the political decisions that followed,[7, 8] particularly given that there was marked cross-national variation in these outcomes.[9] It has been argued that the pursuit of austerity policies in response to the recession, usually involving large-scale public sector reforms, may actually have worsened health outcomes and delayed economic recovery.[10-12] It has also been postulated that austerity policies may worsen inequalities in health outcomes, as they frequently result in cutbacks to programs aiming to address inequitable distribution of the social determinants of health such as housing and education.[13]

The package of austerity measures implemented by the UK Government in 2010 was the third largest in Europe, with substantial cuts especially to welfare, health and social care.[14] Between 2010 and 2015, £26 billion worth of cuts were made to benefits, tax credits, pay and pensions in the UK,[15] with local authorities serving more deprived communities seeing greater financial losses.[16] 85% of financial savings from welfare reforms have been taken from the incomes of women, largely due to the fact that they make up the majority of lone parents and unpaid carers.[17] Women also form a large proportion of the public sector workforce, two-thirds in 2012-13,[18] so are more likely to have been impacted by the two year public sector pay freeze in 2010 and subsequent 1% pay cap that has led to a pay cut in real terms.[14]

Our previous research demonstrated an increase in poor mental health in men but not women following the Great Recession, with no clear evidence for an increase in socioeconomic inequalities.[19]

We aimed for the first time to investigate trends in both gender inequalities and socioeconomic inequalities in poor mental health in the UK following the onset of austerity, and compare these to the immediate aftermath of the 2008 recession.

For peer review only

METHODS

Dataset

Following our previous approach, we used the Health Survey for England (HSE; 1991-2014), a multi-stage stratified random sample designed to be nationally and regionally representative, to construct a repeat cross-sectional dataset. Details of the HSE have been published elsewhere.[20] Response levels have fallen over time but plateaued recently, remaining reasonably high at 62% in 2014 compared with 64% in 2007.[21] Weights for non-response were available from 2003. The rationale for choosing this dataset was the lengthy time period over which it has run using standardised methods, allowing consideration of very long-term trends.

Population

The HSE general population samples were used for all analyses, restricted to those between 25 and 64 years of age to minimise misclassification of employment status among students. Those missing data on age, gender, measure of socioeconomic position (SEP), employment status or outcome were excluded. Sensitivity analysis was performed using the population aged 25-59 years to ensure inclusion of early retirees was not impacting results.

From 1991-2014 there were 128,003 potential participants. 7,774 participants (6.1%) missing outcome data, 109 (0.1%) missing educational attainment, 2964 (2.3%) with foreign or other qualifications which could not be categorised, and 37 (0.03%) missing employment status were excluded, leaving 117,119 participants (91.5%) for inclusion. For analysis using area-level deprivation from 2001 onwards where there were 73,682 potential participants, 5,317 participants (7.2%) missing outcome data, 562 participants (0.7%) missing deprivation score, and 25 (0.03%) missing employment status were excluded, leaving 67,778 participants for inclusion (92.0%).

Exposure Measurement and Covariates

The SEP exposure measures considered were educational attainment and area-level deprivation. Highest educational attainment was available for all years except 1995 and 1996, coded into four categories: degree-level or equivalent, A-level or equivalent, GCSE or equivalent, and no formal qualifications. A marker of small area-level deprivation based on postcode (Index of Multiple Deprivation [IMD] scored in quintiles) was available from 2001.

Covariates considered were employment status and total household income. Employment status was recorded as self-reported activity within the preceding week, coded in six categories: in

For peer review only - http://bmjopen.bmj.com/site/about/guidelines.xhtml

employment, unemployed, retired through ill health, retired, looking after home, or in education. Total household income was available from 1997, coded into quintiles.

The UK economy did not enter recession until the last quarter of 2008 (defined by two successive quarters of negative growth in GDP),[22, 23] and while austerity policies were announced in mid-2010[14] it is unlikely that health consequences would have manifested within this year. We therefore defined in advance all years up to and including 2008 'pre-recession', the years 2009 and 2010 the 'recession period' and from 2012 onwards the 'austerity period' (outcome data were unavailable for 2011).

Outcome Measurement

Poor mental health was assessed using the General Health Questionnaire-12 (GHQ-12), a validated screening tool for common mental health problems used widely in epidemiological research, which scores self-reported symptoms of anxiety and depression.[24] The GHQ-12 formed part of the core questions in each sweep of the HSE except 1996 and 2007, though from 2010 has only been included every second year. A GHQ-12 score of four or greater indicates a strong likelihood of a common mental disorder,[25] and therefore defined a 'case'.

Statistical Analysis

Directly age-standardised prevalence estimates of GHQ caseness were calculated for each year, stratified by gender and both measures of SEP. The 2013 WHO European Standard Population was used for all direct standardisations, and estimates were displayed graphically.

To quantify any potential impact of the recession and austerity on mental health by gender, multivariable logistic regression modelling was performed. First, data from each year were regressed separately to determine long-term trends in the difference between male and female caseness, adjusting for age, education, and employment status. In a combined dataset of all years, models for men and women separately were then created using 2008 as the baseline/pre-recession year, and adjusted for age, SEP, employment status, and total household income. As the main time period of interest was following the point at which IMD was recorded routinely, we focused on this as the primary measure of SEP, given marked changes in the distribution of educational attainment over the study period. In addition to odds ratios, adjusted prevalence differences were derived from the logistic regression models to give a measure of change on the absolute scale.

Long-term trends in socioeconomic inequalities in mental health over time were analysed using the relative index of inequality (RII), a regression-based index comparing the prevalence of the outcome of the theoretically lowest and highest SEP. Analysis was performed using both SEP measures.

For peer review only

Poisson regression was used to generate prevalence risk ratios with 95% confidence intervals,[26] which were then plotted to view trends. All models were adjusted for age and sex.

Patient and Public Involvement

There was no patient or public involvement in the design of this study.

For peer review only

RESULTS

Characteristics of included individuals are displayed in supplementary appendix 1. Over the study period there was a marked increase in women reporting degree-level education, and for both genders the number reporting no formal qualifications fell. During the main time period of interest (2005 onwards) there was little change in gender distribution.

Mental Health Trends by Gender

The prevalence of GHQ caseness was consistently higher in women than men over the study period (Figure 1). There were three clear points of deviation from secular trends for both genders which may be explained by periods of macroeconomic disruption: the late 1990s, early 2000s, and the period following the 2008 recession. The former two may represent the impact of smaller economic downturns during which the UK economy declined but avoided entering recession,[22, 23] and corresponding increases in prevalence were patterned similarly between genders. Conversely, in 2009 there was a marked increase in age-standardised GHQ caseness in men and a more modest increase in women, with only a slight improvement for men in 2010. During the austerity period this patterning altered. While in 2012 male GHQ caseness continued to decrease, female GHQ caseness increased to 18.7% (95% CI 17.2-20.2), its highest observed value since 2002.

[INSERT FIGURE 1]

Between 1991 and 2004 the difference between male and female prevalence, adjusted for age, education and employment status, narrowed from 7.9% (95% CI 4.3-11.4) to 3.3% (95% CI 1.1-5.6). Despite a marked further narrowing of this gender gap in the recession period with a fall to 1.1% (95% CI -1.8-4.0) in 2009, by 2012 it had sharply widened again to 6.8% (95% CI 4.6-8.9), the largest adjusted difference between male and female prevalence since 1994. Values for all years are provided in supplementary appendices 2 and 3.

Table 1 presents logistic regression models from the period of interest for each gender, with tables for the whole time period 2001-2014 available in supplementary appendix 4.

For peer review only

Table 1: Multiple logistic regression models (with odds ratios and % point difference) for participants of each gender, 2005-2014 (2008 as pre-recession reference year)

Regression models for men (n=24,930)												
	Model 1: Adjusted for age, IMD				Model 2: Adjusted for age, IMD, employment				Model 3: Adjusted for age, IMD, employment, income			
Year	Odds Ratio	p-value	Lower 95% CI	Upper 95% CI	Odds Ratio	p-value	Lower 95% CI	Upper 95% CI	Odds Ratio	p-value	Lower 95% CI	Upper 95% CI
2005	1.03	0.722	0.86	1.24	0.92	0.400	0.76	1.12	0.90	0.303	0.74	1.10
2006	1.06	0.476	0.91	1.24	1.04	0.676	0.88	1.22	1.03	0.765	0.87	1.21
2008	1.00	-	-	-	1.00	-	-	-	1.00	-	-	-
2009	1.64	<0.001	1.34	2.00	1.55	<0.001	1.25	1.93	1.53	<0.001	1.24	1.91
2010	1.28	0.009	1.06	1.53	1.26	0.021	1.04	1.52	1.26	0.018	1.04	1.53
2012	1.15	0.147	0.95	1.38	1.10	0.340	0.91	1.33	1.10	0.342	0.91	1.33
2014	1.13	0.215	0.93	1.37	1.17	0.126	0.96	1.43	1.18	0.108	0.96	1.44
Year	% Diff.	p-value	Lower 95% CI	Upper 95% CI	% Diff.	p-value	Lower 95% CI	Upper 95% CI	% Diff.	p-value	Lower 95% CI	Upper 95% CI
2005	0.34	0.723	-1.53	2.20	-0.75	0.396	-2.50	0.99	-0.93	0.298	-2.67	0.82
2006	0.58	0.477	-1.01	2.17	0.33	0.676	-1.21	1.86	0.23	0.765	-1.30	1.77
2008	0.00	-	-	-	0.00	-	-	-	0.00	-	-	-
2009	5.88	<0.001	3.24	8.52	4.72	<0.001	2.20	7.23	4.62	<0.001	2.11	7.12
2010	2.67	0.012	0.60	4.73	2.29	0.024	0.30	4.28	2.37	0.021	0.36	4.38
2012	1.44	0.154	-0.54	3.42	0.89	0.344	-0.95	2.73	0.89	0.346	-0.96	2.74
2014	1.26	0.223	-0.77	3.30	1.53	0.134	-0.47	3.52	1.63	0.115	-0.40	3.65
**Regression models for women (n=31,413)**												
	Model 1: Adjusted for age, IMD				Model 2: Adjusted for age, IMD, employment				Model 3: Adjusted for age, IMD, employment, income			
Year	Odds Ratio	p-value	Lower 95% CI	Upper 95% CI	Odds Ratio	p-value	Lower 95% CI	Upper 95% CI	Odds Ratio	p-value	Lower 95% CI	Upper 95% CI
2005	1.06	0.426	0.92	1.22	1.05	0.531	0.91	1.21	1.02	0.762	0.88	1.18
2006	0.91	0.153	0.81	1.03	0.90	0.102	0.80	1.02	0.89	0.077	0.79	1.01
2008	1.00	-	-	-	1.00	-	-	-	1.00	-	-	-
2009	1.06	0.537	0.89	1.25	1.07	0.429	0.90	1.28	1.07	0.431	0.90	1.28
2010	1.00	0.980	0.86	1.15	0.99	0.849	0.85	1.14	0.99	0.922	0.86	1.15
2012	1.24	0.003	1.08	1.42	1.24	0.003	1.07	1.43	1.25	0.002	1.09	1.45
2014	1.10	0.208	0.95	1.27	1.11	0.171	0.96	1.28	1.12	0.138	0.97	1.29
Year	% Diff.	p-value	Lower 95% CI	Upper 95% CI	% Diff.	p-value	Lower 95% CI	Upper 95% CI	% Diff.	p-value	Lower 95% CI	Upper 95% CI
2005	0.79	0.429	-1.16	2.74	0.60	0.533	-1.29	2.50	0.29	0.762	-1.60	2.18
2006	-1.16	0.153	-2.74	0.43	-1.28	0.102	-2.82	0.25	-1.39	0.077	-2.94	0.15
2008	0.00	-	-	-	0.00	-	-	-	0.00	-	-	-
2009	0.73	0.541	-1.61	3.07	0.93	0.435	-1.40	3.25	0.93	0.436	-1.41	3.27
2010	-0.02	0.98	-1.96	1.91	-0.18	0.848	-2.07	1.70	-0.10	0.922	-2.00	1.81

For peer review only - http://bmjopen.bmj.com/site/about/guidelines.xhtml

2012	3.04	0.004	0.99	5.08	2.90	0.004	0.93	4.88	3.11	0.002	1.11	5.11
2014	1.27	0.213	-0.73	3.28	1.34	0.176	-0.60	3.29	1.47	0.142	-0.49	3.44

GHQ caseness in men was higher in both 2009 and 2010 after adjusting for age and IMD, with prevalence predicted to have increased in the population by 5.9% (95% CI 3.2-8.5, p<0.001) percentage points from 2008 to 2009. This increase remained largely unchanged (4.6%, 95% CI 2.1-7.1, p<0.001) after adjustment for the potential mediating effect of employment status and household income. For men, there was no evidence of significant worsening of population mental health in either 2012 or 2014 when compared with 2008 in any model.

For women, after adjusting for age and IMD there was no evidence of an increase in GHQ caseness during the recessionary period. However, in 2012 the predicted increase in the population compared with 2008 was 3.0% (95% CI 1.0-5.1, p=0.004), and after further adjustment for employment status and household income this remained largely unchanged at 3.1% (95% CI 1.1-5.1, p=0.002). There was a smaller adjusted increase in 2014 compared with 2008 of 1.5% (95% CI -0.5-3.4, p=0.142).

Mental Health Trends by Socioeconomic Position

[INSERT FIGURE 2]

There was a clear socioeconomic gradient in GHQ caseness throughout the study period (Figure 2). The absolute difference between the most and least deprived quintiles was amongst the highest recorded during the austerity period (13.5% in 2012, 11.2% in 2014) compared with smaller differences during the recession period (9.2% in 2009, 8.6% in 2010). All values are provided in supplementary appendix 5.

[INSERT FIGURE 3]

Stratification by highest educational attainment produced similar trends during the recession and austerity periods (Figure 3), with the exception of those with no formal qualifications. This group experienced worsening of GHQ caseness throughout the study period, rising from 15.7% (95% CI 12.8-18.5) in 1991 to 23.7% (95% CI 20.0-27.4) by 2014 without seeing the recovery experienced by other groups during austerity. All values are provided in supplementary appendix 6. To explore the marked worsening for the least educated during austerity, further stratification by gender was performed for the period 2012-2014: the increase in this group was predominantly among men, with age-standardised prevalence rising from 16.0% (95% CI 11.5-20.5) in 2012 to 22.8% (95% CI 17.3-28.3) in 2014, while for women the increase was smaller from 22.7% (95% CI 17.9-27.5) in 2012 to 24.7% (95% CI 19.8-29.5) in 2014.

For peer review only

[INSERT FIGURE 4]

Relative socioeconomic inequalities in GHQ caseness have been consistently observed since 1999 (Figure 4). Inequalities in GHQ caseness have increased from the late 1990s to the immediate pre-recession period, with inequalities generally larger by area-level deprivation. During the recession period there was a slight reduction in socioeconomic inequalities, with RII by education falling from 1.8 (95% CI 1.5-2.2, $p<0.001$) in 2008 to 1.6 (95% CI 1.2-2.1, $p=0.001$) in 2010 and by IMD quintile from 2.3 (95% CI 1.9-2.8, $p<0.001$) in 2008 to 1.9 (95% CI 1.4-2.4, $p<0.001$) in 2010. However, these trends reversed during the austerity period, and by 2014 both RIIs had returned to pre-recession levels. All values are provided in supplementary appendix 7.

For all analyses, sensitivity analysis excluding those aged 60-64 years did not affect trends.

For peer review only

DISCUSSION

In this large repeat cross-sectional study of a representative sample of the English population, we found mental health worsened for women following the onset of austerity policies, while men saw a recovery to pre-recession levels. As a result of the changes, gender inequalities in poor mental health widened during the austerity period, reversing the trend from 1991-2004 of gradual improvement. We also found that socioeconomic inequalities in poor mental health narrowed in the immediate years following the 2008 recession but widened during austerity.

There is conflicting evidence in existing literature around whether mental health inequalities by gender or socioeconomic position have widened in the UK since the recession. Our previous work suggested males saw the sharpest worsening of mental health, and found no evidence of widening socioeconomic inequalities when existing trends were taken into account.[19] However, this was prior to the onset of austerity. More recent evidence showed a more marked worsening of mental health for women in 2014 compared with 2007, but did not take into account intervening years.[27] A large study of pan-European data including the UK found no systematic influence of the recession on socioeconomic inequalities in depression up to 2014,[28] but did not differentiate between the immediate recessionary period and the period following any economic policy response. Work by Barr et al suggested that from 2009 to 2013 there may have been a widening of socioeconomic inequalities in mental health in the UK.[29] However, this used self-reported diagnoses and only two broad categories of socioeconomic group. Our study adds clarity to both areas.

There is no consensus around what factors are responsible for the gender gap in poor mental health. There is little evidence it results from purely genetic or biological differences, with sociocultural roles, adverse life events and learned psychological attributes thought more likely contributing factors.[30] Our findings of a reversal in trend direction echo those of others who have begun to raise concerns about the mental health of UK women in recent years, particularly young women.[27, 31] The timing of this reversal in relation to austerity reforms, and the differential gender patterning of austerity,[17] could indicate that the change for women may be secondary to the policy response rather than the economic crisis itself.

The finding of a reversal in trend towards widening socioeconomic inequalities following the onset of austerity adds to the evidence base arguing such measures may mediate the link between macroeconomic change and mental health.[2, 10] Ecological studies using pan-European data suggest the direct effect of unemployment on suicide rates was greater in countries with lower social spending,[32] and conversely, higher government spending on unemployment support may mitigate adverse impacts on self-rated health.[33] On a relative scale the widening of socioeconomic

For peer review only

inequalities post-austerity is small in the context of long-term trends, possibly explaining the current lack of consensus.[28, 29]

The marked divergence for those with no formal qualifications by 2014 may support the hypothesis that those in low-skilled jobs (who are known to experience poorer health outcomes[34]) may be worst affected by reduced in-work financial support or worsening job conditions such as increased insecure work.[35] Their divergence may also be partly attributable to changes in demographics over the study period, with the group achieving no qualifications becoming smaller and more homogenous over time. Regardless, they are notable outliers in 2014, identifying this group as particularly high risk for poor mental health.

Strengths and Limitations

Our study has a number of important strengths. The HSE is a large, nationally and regionally representative survey which has used standard protocols over a long period of time. The GHQ-12 is a validated and commonly used measure, and outcome data were available for most years allowing detailed consideration of trends. The use and comparison of two measures of SEP is useful in demonstrating consistency of trends between SEP and poor mental health.

Our study also has some limitations which must be considered. The use of cross-sectional rather than individual longitudinal data mean the ability to derive causal inferences is limited; however, it does overcome attrition bias in cohort studies which can commonly lead to an underestimation of inequalities.[36] As data were not collected on whether individuals were subject to specific austerity measures, this could not be included as an explanatory variable. Household income was felt to be a reasonable proxy given that most reforms were associated with financial loss.[16] It is unfortunate that outcome data were not available from 2007, 2011, 2013 or 2015, as this would have strengthened the evidence for the assessment of trends.

Further research using longitudinal data would add strength to any argument for causality, as would replication using alternative outcome measures, such as antidepressant prescriptions. Distinguishing between the impact of different components of austerity measures, e.g. public sector employment terms, welfare reforms, or access to community services, could add further nuance to our reporting of their potential combined impact. Furthermore, increasing devolution provides the opportunity to study differences in policy approaches within the UK.[37] Cross-national comparisons would also be useful in determining whether observed trends are replicated elsewhere, and whether impacts are dependent on levels of austerity, and natural experiment approaches could strengthen causal inference.[38] Finally, it is clearly important to see whether the observed trajectories in mental

For peer review only

health inequalities have continued following 2014, particularly given that more severe welfare reforms were initiated in 2015.[16]

Conclusions

This study adds to what the European Psychiatric Association in 2016 described as an emerging 'broad consensus about the deleterious consequences of economic crises on mental health'.[39] The gender gap in mental health, which had been improving prior to the recession, appears to be sharply widening again following the onset of austerity policies which have largely focused on women. Those in the most deprived groups have been shown to be at potentially heightened risk of poor mental health following the onset of austerity, with the least educated at highest risk.

These findings are alarming, particularly given that since the time period studied there have been further cuts to mental health provision which mean the issue may now be worse.[40] Labonté and Stuckler argue in strong terms that, based on current evidence of economic, health and social harms, austerity policies threaten to 'imperil the world's population' without radical reform.[2] Policymakers in the UK, and those considering embarking on or continuing austerity measures elsewhere in the world, should be aware that these may have adverse health impacts for their populations.

For peer review only

DECLARATIONS

Acknowledgements

We would like to thank Frank Popham for providing feedback on drafts of the manuscript. We are extremely grateful to the individuals who took part in all cycles of the Health Survey for England, and all those involved in its administration over the years. We would like to acknowledge the assistance of the UK Data Archive for providing access to the data; the Information Centre for health and social care and Department of Health for sponsoring the Health Survey for England; and the Principal Investigators of the Health Survey for England, Natcen Social Research and the Department of Epidemiology and Public Health at the Royal Free and University College Medical School. The responsibility for the analysis presented here lies solely with the authors.

Competing Interests

The authors declare no conflicts of interest.

Funding

SVK is funded by a NRS Senior Clinical Fellowship (SCAF/15/02), the Medical Research Council (MC_UU_12017/13 & MC_UU_12017/15) and Scottish Government Chief Scientist Office (SPHSU13 & SPHSU15). CLN is funded by the Medical Research Council (MR/R024774/1).

Contributors

RT serves as guarantor for this article. RT and SVK conceived the idea for the study, and RT performed the statistical analysis and wrote the first draft of the article. CN, FP and SVK assisted in research design, interpretation of findings and critical revision of the manuscript.

Ethics Approval

Ethical approval was not required for this study as it used previously collected data. Ethical approval for each year of the survey was obtained by the Health Survey for England team.

Data Sharing Statement

Supplementary files are provided.

For peer review only

REFERENCES

1. Stuckler D, Basu S, Suhrcke M, et al. The public health effect of economic crises and alternative policy responses in Europe: an empirical analysis. *Lancet* 2009;374(9686):315-23. doi: 10.1016/S0140-6736(09)61124-7

2. Labonte R, Stuckler D. The rise of neoliberalism: how bad economics imperils health and what to do about it. *J Epidemiol Community Health* 2016;70(3):312-18. doi: 10.1136/jech-2015-206295

3. Frasquilho D, Matos MG, Salonna F, et al. Mental health outcomes in times of economic recession: a systematic literature review. *BMC Public Health* 2016;16:115. doi: 10.1186/s12889-016-2720-y [published Online First: 2016/02/06]

4. Katikireddi SV, Niedzwiedz CL, Popham F. Employment status and income as potential mediators of educational inequalities in population mental health. *Eur J Public Health* 2016;26(5):814-16. doi: 10.1093/eurpub/ckw126

5. Tapia Granados JA, Ionides EL. Population health and the economy: Mortality and the Great Recession in Europe. *Health Econ* 2017 doi: 10.1002/hec.3495 [published Online First: 2017/03/28]

6. Toffolutti V, Suhrcke M. Assessing the short term health impact of the Great Recession in the European Union: a cross-country panel analysis. *Prev Med* 2014;64:54-62. doi: 10.1016/j.ypmed.2014.03.028 [published Online First: 2014/04/11]

7. Karanikolos M, Heino P, McKee M, et al. Effects of the Global Financial Crisis on Health in High-Income Oecd Countries: A Narrative Review. *Int J Health Serv* 2016;46(2):208-40. doi: 10.1177/0020731416637160

8. Stuckler D, Basu S. The Body Economic: Eight experiments in economic recovery, from Iceland to Greece. London: Penguin 2013.

9. Reeves A, McKee M, Stuckler D. Economic suicides in the Great Recession in Europe and North America. *Br J Psychiatry* 2014;205(3):246-7. doi: 10.1192/bjp.bp.114.144766 [published Online First: 2014/06/14]

10. Karanikolos M, Mladovsky P, Cylus J, et al. Financial crisis, austerity, and health in Europe. *Lancet* 2013;381(9874):1323-31. doi: 10.1016/s0140-6736(13)60102-6 [published Online First: 2013/04/02]

11. De Vogli R. The financial crisis, health and health inequities in Europe: the need for regulations, redistribution and social protection. *Int J Equity Health* 2014;13:58. doi: 10.1186/s12939-014-0058-6 [published Online First: 2014/07/26]

For peer review only

12. Loopstra R, McKee M, Katikireddi SV, et al. Austerity and old-age mortality in England: a longitudinal cross-local area analysis, 2007-2013. *J R Soc Med* 2016;109(3):109-16. doi: 10.1177/0141076816632215

13. Ruckert A, Labonte R. The global financial crisis and health equity: early experiences from Canada. *Global Health* 2014;10:2. doi: 10.1186/1744-8603-10-2 [published Online First: 2014/01/08]

14. Reeves A, Basu S, McKee M, et al. Austere or not? UK coalition government budgets and health inequalities. *J R Soc Med* 2013;106(11):432-6. doi: 10.1177/0141076813501101

15. Engender, Scottish Refugee Council, Scottish Women's Aid, et al. A Widening Gap: Women and Welfare Reform. Scotland: Engender, 2015.

16. Beatty C, Fothergill S. The uneven impact of welfare reform: The financial losses to places and people. Sheffield: Centre for Regional Economic and Social Research, Sheffield Hallam University, 2016.

17. Ariss A, Firmin C, Meacher B, et al. Where's the Benefit? An Independent Inquiry into Women and Jobseeker's Allowance. In: Society TF, ed. London: The Fawcett Society, 2015.

18. Cribb J, Disney R, Sibieta L. The public sector workforce: past, present and future. London, 2014.

19. Katikireddi SV, Niedzwiedz CL, Popham F. Trends in population mental health before and after the 2008 recession: a repeat cross-sectional analysis of the 1991-2010 Health Surveys of England. *BMJ Open* 2012;2(5) doi: 10.1136/bmjopen-2012-001790

20. Mindell J, Biddulph JP, Hirani V, et al. Cohort profile: the health survey for England. *Int J Epidemiol* 2012;41(6):1585-93. doi: 10.1093/ije/dyr199 [published Online First: 2012/01/19]

21. NatCen Social Research, University College London, Department of Epidemiology and Public Health. Health Survey for England: UK Data Service, 1991-2014.

22. Macrotrends LLC. FTSE 100 Index - 32 Year Historical Chart [Online]: Macrotrends LLC; [updated June 2017; cited 23rd June 2017]. Available from: http://www.macrotrends.net/2598/ftse-100-index-historical-chart-data.

23. The Office for National Statistics. Times Series: Gross Domestic Product: Quarter on Quarter Growth: CVM SA%: ONS; [updated May 2017; cited 30th June 2017]. Available from: https://www.ons.gov.uk/economy/grossdomesticproductgdp/timeseries/ihyq/pn2.

24. Goldberg DP, Gater R, Sartorius N, et al. The validity of two versions of the GHQ in the WHO study of mental illness in general health care. *Psychol Med* 1997;27(1):191-7. [published Online First: 1997/01/01]

For peer review only

25. Goldberg DP, Williams, P. A user's guide to the General Health Questionnaire. Windsor: NFER-Nelson 1988.

26. Mood C. Logistic Regression: Why We Cannot Do What We Think We Can Do, and What We Can Do About It. *Eur Sociol Rev* 2010;26(1):67-82.

27. McManus S, Bebbington P, Jenkins R, et al. Mental health and wellbeing in England: Adult Psychiatric Morbidity Survey 2014. Leeds: NHS Digital 2016.

28. Reibling N, Beckfield J, Huijts T, et al. Depressed during the depression: has the economic crisis affected mental health inequalities in Europe? Findings from the European Social Survey (2014) special module on the determinants of health. *Eur J Public Health* 2017;27(suppl_1):47-54. doi: 10.1093/eurpub/ckw225 [published Online First: 2017/03/30]

29. Barr B, Kinderman P, Whitehead M. Trends in mental health inequalities in England during a period of recession, austerity and welfare reform 2004 to 2013. *Soc Sci Med* 2015;147:324-31. doi: 10.1016/j.socscimed.2015.11.009

30. Piccinelli M, Wilkinson G. Gender differences in depression. Critical review. *Br J Psychiatry* 2000;177:486-92. [published Online First: 2000/12/05]

31. Lessof C, Ross A, Brind R, et al. Longitudinal Study of Young People in England cohort 2: health and wellbeing at wave 2; Research report UK: TNS BMRB, 2016.

32. Baumbach A, Gulis G. Impact of financial crisis on selected health outcomes in Europe. *Eur J Public Health* 2014;24(3):399-403. doi: 10.1093/eurpub/cku042 [published Online First: 2014/04/09]

33. Toge AG. Health Effects of Unemployment in Europe During the Great Recession: The Impact of Unemployment Generosity. *Int J Health Serv* 2016;46(4):614-41. doi: 10.1177/0020731416664688 [published Online First: 2016/08/27]

34. Katikireddi SV, Leyland AH, McKee M, et al. Patterns of mortality by occupation in the UK, 1991-2011: a comparative analysis of linked census and mortality records. *Lancet Public Health* 2017;2(11):e501-e12. doi: 10.1016/S2468-2667(17)30193-7

35. Benach J, Vives A, Amable M, et al. Precarious employment: understanding an emerging social determinant of health. *Annu Rev Public Health* 2014;35:229-53. doi: 10.1146/annurev-publhealth-032013-182500

36. Howe LD, Tilling K, Galobardes B, et al. Loss to follow-up in cohort studies: bias in estimates of socioeconomic inequalities. *Epidemiology* 2013;24(1):1-9. doi: 10.1097/EDE.0b013e31827623b1 [published Online First: 2012/12/06]

For peer review only

37. Katikireddi SV, Smith KE, Stuckler D, et al. Devolution of power, revolution in public health? *J Public Health (Oxf)* 2017;39(2):241-47. doi: 10.1093/pubmed/fdw031

38. Craig P, Katikireddi SV, Leyland A, et al. Natural Experiments: An Overview of Methods, Approaches, and Contributions to Public Health Intervention Research. *Annu Rev Public Health* 2017;38:39-56. doi: 10.1146/annurev-publhealth-031816-044327

39. Martin-Carrasco M, Evans-Lacko S, Dom G, et al. EPA guidance on mental health and economic crises in Europe. *Eur Arch Psychiatry Clin Neurosci* 2016;266(2):89-124. doi: 10.1007/s00406-016-0681-x [published Online First: 2016/02/15]

40. British Medical Association. Mental health budgets cut [Online] [updated February 2017; cited October 2017]. Available from: https://www.bma.org.uk/news/2017/february/mental-health-budgets-cut.

For peer review only

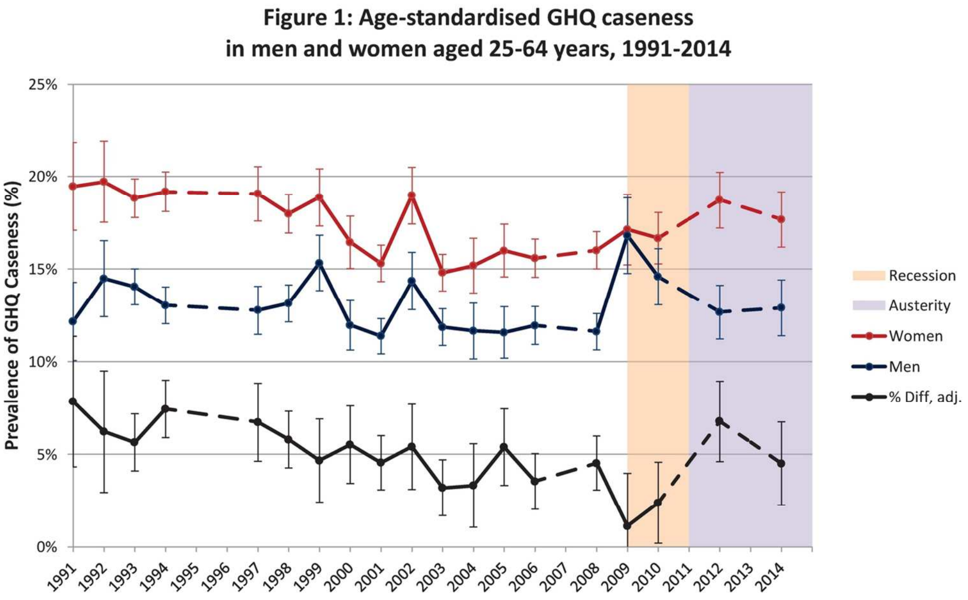

130x80mm (300 x 300 DPI)

Figure 2: Age-sex standardised GHQ caseness by IMD quintile in 25-64 year olds, 2001-2014

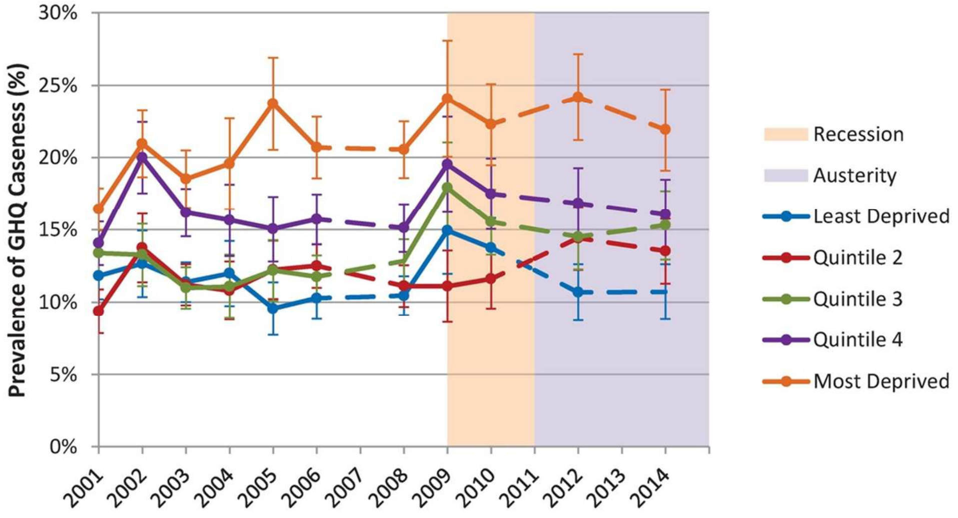

99x63mm (300 x 300 DPI)

view only

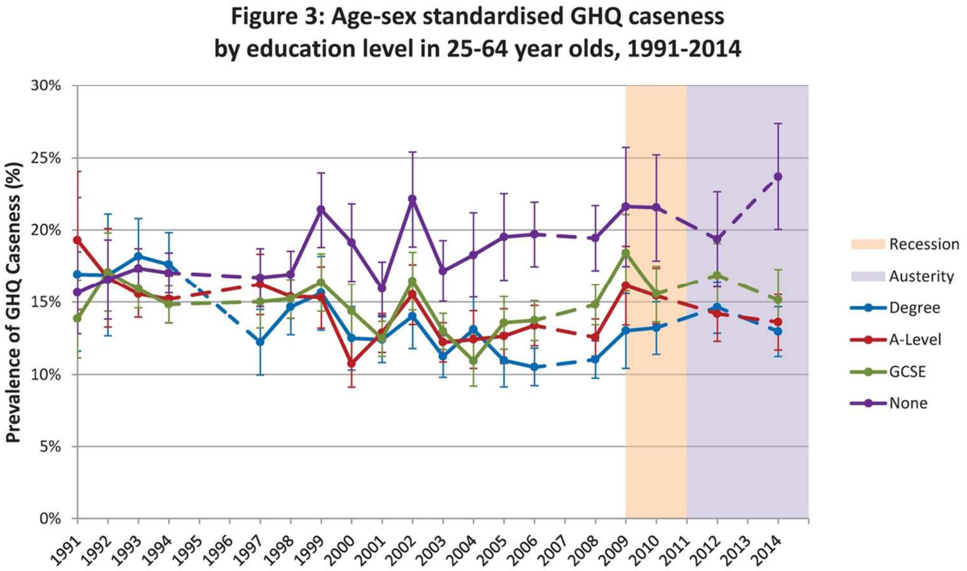

119x71mm (300 x 300 DPI)

For peer review only

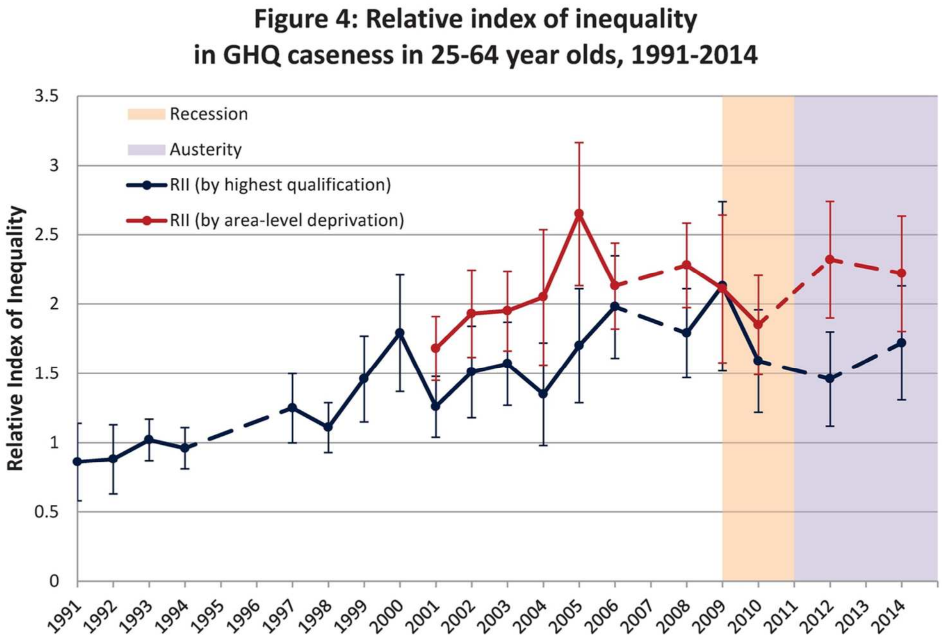

119x79mm (300 x 300 DPI)

view only

Supplementary Appendix

Appendix 1: Characteristics of study participants

		Gender (%)	Age Group (%)				Highest Education Level (%)					
**Year**			**25-34**	**35-44**	**45-54**	**55-64**	**Degree**	**A-Level**	**GCSE**	**None**	**Sample**	**Total Sample**
**1991**	Male	46.6	29.7	27.6	21.9	20.8	16.0	20.1	32.1	31.9	942	
	Female	53.4	30.0	27.2	21.7	21.2	8.1	17.3	34.0	40.6	1078	**2020**
**1992**	Male	48.4	28.6	28.4	23.8	19.2	15.2	26.0	32.5	26.3	1184	
	Female	51.6	29.8	28.4	21.7	20.1	8.7	18.5	37.9	34.9	1316	**2500**
**1993**	Male	47.6	29.2	26.1	24.9	19.8	16.4	25.4	30.1	28.2	5030	
	Female	52.4	30.2	27.0	23.8	19.0	9.6	18.2	35.9	36.2	5544	**10,574**
**1994**	Male	46.7	29.5	27.3	23.0	20.2	15.9	26.5	30.0	27.5	4704	
	Female	53.3	31.0	27.2	23.1	18.8	10.4	18.0	37.9	33.7	5361	**10,065**
**1997**	Male	47.2	27.7	27.4	25.5	19.4	19.2	28.7	28.3	23.8	2559	
	Female	52.8	30.6	27.2	24.5	17.7	14.0	21.3	33.7	31.0	2860	**5419**
**1998**	Male	46.6	27.7	26.9	25.8	19.6	19.5	28.0	29.5	23.1	4582	
	Female	53.4	29.0	28.0	24.7	18.3	14.3	20.6	36.0	29.2	5254	**9836**
**1999**	Male	47.0	25.4	28.1	25.4	21.0	21.0	28.5	27.5	23.0	2257	
	Female	53.0	27.4	29.9	25.7	17.0	15.5	21.6	34.4	28.6	2543	**4800**
**2000**	Male	45.8	25.9	29.7	23.4	21.0	21.7	30.3	27.0	21.0	2311	
	Female	54.2	27.6	30.5	23.3	18.5	16.5	25.0	34.1	24.4	2733	**5044**
**2001**	Male	45.6	24.2	28.3	25.6	21.9	22.7	29.2	29.4	18.7	4360	
	Female	54.4	25.6	30.7	25.0	18.7	17.2	23.6	35.3	23.9	5193	**9553**
**2002**	Male	43.4	23.0	31.5	23.8	21.7	23.1	31.6	28.2	17.2	2016	
	Female	56.6	27.8	32.1	22.0	18.2	19.4	25.1	36.2	19.3	2633	**4649**
**2003**	Male	45.5	22.6	28.6	24.4	24.3	23.6	28.0	29.7	18.7	4117	
	Female	54.6	23.9	30.6	23.3	22.1	20.4	24.0	34.8	20.8	4941	**9058**

For Peer review only

2004	Male	43.4	23.2	27.7	22.8	26.3	25.4	27.7	27.1	19.9	1782	
	Female	56.6	21.8	30.3	24.3	23.5	21.1	24.3	31.9	22.8	2327	**4109**
2005	Male	44.8	22.0	25.0	26.9	26.0	25.5	29.0	26.7	18.8	2076	
	Female	55.2	22.9	27.9	26.1	23.2	22.1	23.4	32.6	21.9	2555	**4631**
2006	Male	44.8	19.9	28.2	25.0	26.9	27.2	29.9	25.6	17.3	3896	
	Female	55.2	22.2	29.2	24.7	23.8	24.2	24.3	32.1	19.4	4795	**8691**
2008	Male	44.8	21.1	27.2	24.8	26.9	27.2	30.1	25.9	16.8	4165	
	Female	55.2	22.7	27.9	25.4	24.1	24.5	26.3	30.9	18.3	5135	**9300**
2009	Male	45.6	20.7	27.8	25.9	25.7	27.4	26.8	29.3	16.5	1278	
	Female	54.5	22.6	29.7	24.2	23.4	25.9	24.4	32.7	17.2	1528	**2806**
2010	Male	43.5	20.3	26.1	26.7	26.8	29.6	30.2	26.6	13.6	2123	
	Female	56.5	22.1	26.8	28.2	22.9	27.5	26.6	32.0	13.9	2759	**4882**
2012	Male	43.6	19.8	26.1	26.8	27.4	32.2	29.8	24.3	13.7	2025	
	Female	56.5	22.9	26.1	28.2	22.9	32.3	27.1	26.5	14.1	2625	**4650**
2014	Male	43.3	19.2	26.7	29.8	24.3	34.2	27.9	24.6	13.4	1964	
	Female	56.7	22.7	27.6	28.3	21.4	33.6	28.3	25.8	12.4	2568	**4532**

		Gender (%)	Age Group (%)				IMD Quintile						
**Year**			**25-34**	**35-44**	**45-54**	**55-64**	**1 (Least Deprived)**	**2**	**3**	**4**	**5 (Most Deprived)**	**Sample**	**Total Sample**
2001	Male	44.8	24.0	27.8	26.1	22.2	16.7	15.2	19.2	21.0	28.0	4211	
	Female	55.2	24.8	29.6	25.6	20.1	16.4	14.8	20.0	22.1	26.8	5184	**9395**
2002	Male	42.7	22.9	31.1	23.9	22.1	16.6	16.6	18.6	22.2	26.0	2080	
	Female	57.3	26.8	31.0	22.7	19.6	17.2	16.0	19.0	21.6	26.2	2794	**4874**
2003	Male	44.6	22.7	28.4	24.5	24.5	22.6	20.8	19.9	20.7	16.1	4204	
	Female	55.4	23.1	29.7	23.4	23.8	23.2	20.1	19.8	20.7	16.2	5229	**9433**
2004	Male	42.9	23.2	27.7	22.8	26.3	23.4	23.2	19.1	20.3	14.1	1786	
	Female	57.1	21.3	29.8	24.3	24.6	22.3	22.8	17.8	19.9	17.2	2380	**4166**

For peer review only

**2005**	Male	44.3	22.0	25.0	26.9	26.2	23.2	21.3	20.5	20.6	14.5	2082	
	Female	55.7	22.4	27.4	25.9	24.3	22.2	21.6	19.1	20.7	16.4	2618	**4700**
**2006**	Male	44.4	19.8	28.2	25.0	26.9	20.9	21.4	21.4	19.8	16.5	3907	
	Female	55.6	22.0	28.8	24.6	24.7	20.2	22.7	21.0	19.4	16.7	4888	**8795**
**2008**	Male	44.4	21.1	27.2	24.7	27.0	22.3	20.3	19.9	19.4	18.1	4169	
	Female	55.6	22.3	27.5	25.2	25.0	22.6	19.9	20.0	19.2	18.3	5230	**9399**
**2009**	Male	45.1	20.6	27.8	25.8	25.8	20.4	22.4	20.4	20.0	16.9	1282	
	Female	54.9	22.3	29.3	24.3	24.2	20.6	21.4	21.2	20.0	16.7	1559	**2841**
**2010**	Male	43.1	20.3	26.1	26.7	26.9	23.4	19.8	20.0	19.0	17.9	2129	
	Female	56.9	21.9	26.6	28.1	23.6	23.7	19.7	20.0	19.5	17.3	2806	**4935**
**2012**	Male	43.4	19.8	26.1	26.9	27.3	22.1	21.1	20.2	19.3	17.4	2030	
	Female	56.6	22.8	25.9	28.0	23.4	22.7	20.7	19.9	19.2	17.6	2649	**4679**
**2014**	Male	43.2	19.3	26.6	29.9	24.3	23.5	20.3	18.4	18.7	19.1	1969	
	Female	56.8	22.6	27.6	28.1	21.8	21.7	19.6	20.0	19.7	19.1	2592	**4561**

For peer review only

Appendix 2: Age-standardised GHQ caseness in men and women aged 25-64 years, 1991-2014

(displayed in Figure 1 in main text)

	Men				Women			
**Year**	Sample Size	Adjusted Est. (%)	Lower 95% CI	Upper 95% CI	Sample Size	Adjusted Est. (%)	Lower 95% CI	Upper 95% CI
**1991**	942	12.17	10.06	14.28	1078	19.46	17.08	21.84
**1992**	1184	14.48	12.44	16.52	1316	19.71	17.52	21.90
**1993**	5030	14.04	13.07	15.01	5544	18.82	17.78	19.86
**1994**	4704	13.04	12.06	14.02	5361	19.17	18.10	20.24
**1997**	2559	12.77	11.47	14.07	2860	19.06	17.60	20.52
**1998**	4582	13.14	12.15	14.13	5254	17.98	16.94	19.03
**1999**	2257	15.32	13.83	16.81	2543	18.87	17.33	20.41
**2000**	2311	11.97	10.63	13.30	2733	16.44	15.03	17.85
**2001**	4360	11.38	10.43	12.32	5193	15.30	14.31	16.28
**2002**	2016	14.35	12.81	15.89	2633	18.97	17.44	20.50
**2003**	4117	11.86	10.87	12.85	4941	14.80	13.81	15.80
**2004**	1782	11.66	10.16	13.17	2327	15.18	13.71	16.64
**2005**	2076	11.57	10.19	12.94	2555	15.99	14.57	17.41
**2006**	3896	11.95	10.93	12.98	4795	15.58	14.55	16.61
**2008**	4165	11.62	10.64	12.60	5135	16.01	15.01	17.02
**2009**	1278	16.80	14.75	18.86	1528	17.13	15.23	19.04
**2010**	2123	14.58	13.07	16.09	2759	16.66	15.27	18.05
**2012**	2025	12.67	11.22	14.11	2625	18.72	17.22	20.21
**2014**	1964	12.90	11.40	14.40	2568	17.67	16.19	19.15

For peer review only

Appendix 3: Adjusted odds ratio and % point difference in GHQ caseness by gender, 1991-2014

(displayed in Figure 1 in main text)

	Model 1: Adjusted for age			Model 2: Adjusted for age, education, employment		
**Year**	**OR (95% CI)**	**p-value**	**% Diff. (95% CI)**	**OR (95% CI)**	**p-value**	**% Diff. (95% CI)**
**1991**	1.76 (1.38-2.26)	<0.001	7.61 (4.31-10.90)	1.83 (1.39-2.40)	<0.001	7.85 (4.33-11.37)
**1992**	1.48 (1.20-1.83)	<0.001	5.53 (2.53-8.52)	1.57 (1.24-1.98)	<0.001	6.23 (2.95-9.50)
**1993**	1.43 (1.28-1.58)	<0.001	4.91 (3.47-6.35)	1.52 (1.35-1.70)	<0.001	5.64 (4.11-7.17)
**1994**	1.60 (1.44-1.79)	<0.001	6.42 (4.94-7.89)	1.76 (1.57-1.98)	<0.001	7.44 (5.89-9.00)
**1997**	1.60 (1.38-1.85)	<0.001	6.31 (4.31-8.31)	1.68 (1.43-1.98)	<0.001	6.73 (4.63-8.84)
**1998**	1.47 (1.32-1.64)	<0.001	5.11 (3.65-6.58)	1.58 (1.40-1.78)	<0.001	5.79 (4.26-7.33)
**1999**	1.29 (1.11-1.51)	0.001	3.68 (1.52-5.83)	1.41 (1.20-1.67)	<0.001	4.67 (2.42-6.91)
**2000**	1.45 (1.23-1.70)	<0.001	4.56 (2.58-6.54)	1.62 (1.35-1.95)	<0.001	5.52 (3.43-7.61)
**2001**	1.41 (1.25-1.59)	<0.001	4.04 (2.64-5.45)	1.51 (1.32-1.72)	<0.001	4.55 (3.10-6.01)
**2002**	1.41 (1.20-1.65)	<0.001	4.77 (2.55-6.98)	1.50 (1.26-1.79)	<0.001	5.41 (3.11-7.71)
**2003**	1.27 (1.12-1.44)	<0.001	2.74 (1.30-4.17)	1.34 (1.17-1.54)	<0.001	3.19 (1.69-4.69)
**2004**	1.33 (1.10-1.60)	0.004	3.22 (1.07-5.37)	1.36 (1.10-1.69)	0.004	3.32 (1.06-5.57)
**2005**	1.49 (1.25-1.78)	<0.001	4.68 (2.67-6.70)	1.65 (1.36-2.01)	<0.001	5.39 (3.32-7.45)
**2006**	1.32 (1.16-1.50)	<0.001	3.23 (1.73-4.72)	1.39 (1.21-1.60)	<0.001	3.54 (2.03-5.05)
**2008**	1.45 (1.28-1.64)	<0.001	4.35 (2.91-5.79)	1.53 (1.33-1.75)	<0.001	4.53 (3.07-5.99)
**2009**	0.98 (0.80-1.21)	0.866	-0.25 (-3.12-2.62)	1.09 (0.87-1.37)	0.446	1.12 (-1.75-3.98)
**2010**	1.17 (0.99-1.39)	0.065	2.08 (-0.11-4.27)	1.22 (1.01-1.46)	0.034	2.39 (0.19-4.58)
**2012**	1.57 (1.32-1.86)	<0.001	5.93 (3.70-8.15)	1.79 (1.48-2.16)	<0.001	6.78 (4.61-8.94)
**2014**	1.42 (1.19-1.70)	<0.001	4.53 (2.28-6.78)	1.43 (1.19-1.71)	<0.001	4.51 (2.27-6.75)

For peer review only

Appendix 4: Full multiple logistic regression models for participants of each gender, 2001-2014

Regression models for men (n=24,930)												
	Model 1: Adjusted for age, IMD				Model 2: Adjusted for age, IMD, employment				Model 3: Adjusted for age, IMD, employment, income			
**Year**	**Odds Ratio**	**p-value**	**Lower 95% CI**	**Upper 95% CI**	**Odds Ratio**	**p-value**	**Lower 95% CI**	**Upper 95% CI**	**Odds Ratio**	**p-value**	**Lower 95% CI**	**Upper 95% CI**
**2001**	0.93	0.330	0.80	1.08	0.89	0.147	0.76	1.04	0.86	0.069	0.73	1.01
**2002**	1.27	0.007	1.07	1.51	1.29	0.006	1.07	1.54	1.26	0.014	1.05	1.51
**2003**	1.06	0.481	0.91	1.23	0.97	0.667	0.82	1.13	0.94	0.475	0.80	1.11
**2004**	1.11	0.272	0.92	1.35	1.07	0.505	0.87	1.31	1.05	0.646	0.86	1.29
**2005**	1.03	0.722	0.86	1.24	0.92	0.400	0.76	1.12	0.90	0.303	0.74	1.10
**2006**	1.06	0.476	0.91	1.24	1.04	0.676	0.88	1.22	1.03	0.765	0.87	1.21
**2008**	1.00	-	-	-	1.00	-	-	-	1.00	-	-	-
**2009**	1.64	<0.001	1.34	2.00	1.55	<0.001	1.25	1.93	1.53	<0.001	1.24	1.91
**2010**	1.28	0.009	1.06	1.53	1.26	0.021	1.04	1.52	1.26	0.018	1.04	1.53
**2012**	1.15	0.147	0.95	1.38	1.10	0.340	0.91	1.33	1.10	0.342	0.91	1.33
**2014**	1.13	0.215	0.93	1.37	1.17	0.126	0.96	1.43	1.18	0.108	0.96	1.44
**Regression models for women (n=31,413)**												
	Model 1: Adjusted for age, IMD				Model 2: Adjusted for age, IMD, employment				Model 3: Adjusted for age, IMD, employment, income			
**Year**	**Odds Ratio**	**p-value**	**Lower 95% CI**	**Upper 95% CI**	**Odds Ratio**	**p-value**	**Lower 95% CI**	**Upper 95% CI**	**Odds Ratio**	**p-value**	**Lower 95% CI**	**Upper 95% CI**
**2001**	0.94	0.280	0.83	1.05	0.91	0.143	0.81	1.03	0.87	0.030	0.78	0.99
**2002**	1.14	0.049	1.00	1.31	1.13	0.078	0.99	1.30	1.10	0.162	0.96	1.27
**2003**	0.92	0.192	0.82	1.04	0.91	0.112	0.80	1.02	0.87	0.026	0.77	0.98
**2004**	0.93	0.358	0.80	1.08	0.91	0.218	0.78	1.06	0.88	0.112	0.75	1.03
**2005**	1.06	0.426	0.92	1.22	1.05	0.531	0.91	1.21	1.02	0.762	0.88	1.18
**2006**	0.91	0.153	0.81	1.03	0.90	0.102	0.80	1.02	0.89	0.077	0.79	1.01
**2008**	1.00	-	-	-	1.00	-	-	-	1.00	-	-	-
**2009**	1.06	0.537	0.89	1.25	1.07	0.429	0.90	1.28	1.07	0.431	0.90	1.28
**2010**	1.00	0.980	0.86	1.15	0.99	0.849	0.85	1.14	0.99	0.922	0.86	1.15
**2012**	1.24	0.003	1.08	1.42	1.24	0.003	1.07	1.43	1.25	0.002	1.09	1.45
**2014**	1.10	0.208	0.95	1.27	1.11	0.171	0.96	1.28	1.12	0.138	0.97	1.29

Appendix 5: Age-sex standardised GHQ caseness by IMD quintile in 25–64-year-olds, 2001-2014
(displayed in Figure 2 in main text)

Year	Quintile	Sample Size	Adjusted Est. (%)	Lower 95% CI	Upper 95% CI	Year	Quintile	Sample Size	Adjusted Est. (%)	Lower 95% CI	Upper 95% CI
**2001**	Least Dep.	1549	11.80	10.20	13.41	**2002**	Least Dep.	825	12.65	10.35	14.94
	2	1403	9.36	7.83	10.88		2	794	13.74	11.37	16.11
	3	1846	13.38	11.83	14.94		3	916	13.26	11.11	15.42
	4	2030	14.06	12.56	15.57		4	1065	19.99	17.52	22.46
	Most Dep.	2567	16.42	14.97	17.88		Most Dep.	1274	20.94	18.64	23.24
**2003**	Least Dep.	2163	11.38	10.00	12.75	**2004**	Least Dep.	948	11.98	9.73	14.22
	2	1924	11.19	9.78	12.61		2	957	10.79	8.78	12.80
	3	1872	10.98	9.55	12.40		3	764	11.07	8.87	13.28
	4	1950	16.18	14.54	17.83		4	836	15.66	13.18	18.13
	Most Dep.	1524	18.51	16.53	20.49		Most Dep.	661	19.56	16.43	22.70
**2005**	Least Dep.	1064	9.55	7.73	11.37	**2006**	Least Dep.	1806	10.27	8.81	11.73
	2	1008	12.23	10.22	14.24		2	1943	12.50	11.00	13.99
	3	927	12.18	10.08	14.27		3	1864	11.75	10.28	13.21
	4	970	15.05	12.81	17.28		4	1719	15.71	13.97	17.45
	Most Dep.	731	23.72	20.53	26.92		Most Dep.	1463	20.68	18.56	22.80
**2008**	Least Dep.	2108	10.44	9.10	11.78	**2009**	Least Dep.	582	14.92	11.95	17.90
	2	1889	11.11	9.67	12.54		2	621	11.09	8.62	13.57
	3	1877	12.83	11.33	14.33		3	593	17.91	14.79	21.03
	4	1815	15.12	13.47	16.76		4	568	19.51	16.21	22.81
	Most Dep.	1710	20.54	18.59	22.50		Most Dep.	477	24.07	20.06	28.08
**2010**	Least Dep.	1162	13.73	11.66	15.81	**2012**	Least Dep.	1048	10.67	8.72	12.62
	2	973	11.60	9.55	13.65		2	977	14.40	12.22	16.58
	3	985	15.53	13.28	17.78		3	937	14.50	12.26	16.75
	4	951	17.48	15.05	19.92		4	899	16.83	14.40	19.26
	Most Dep.	864	22.28	19.47	25.09		Most Dep.	818	24.18	21.21	27.16
**2014**	Least Dep.	1024	10.71	8.80	12.62						
	2	907	13.52	11.27	15.76						
	3	880	15.30	12.93	17.67						
	4	879	16.04	13.61	18.48						
	Most Dep.	871	21.91	19.10	24.73						

Appendix 6: Age-sex standardised GHQ caseness by education level in 25–64-year-olds, 1991-2014

(displayed in Figure 3 in main text)

Year	Educ. Level	Sample Size	Adjusted Est. (%)	Lower 95% CI	Upper 95% CI	Year	Educ. Level	Sample Size	Adjusted Est. (%)	Lower 95% CI	Upper 95% CI
**1991**	Degree	238	16.92	11.61	22.24	**1992**	Degree	295	16.88	12.67	21.09
	A-Level	375	19.29	14.49	24.08		A-Level	551	16.69	13.28	20.10
	GCSE	669	13.85	11.15	16.54		GCSE	884	17.08	14.36	19.79
	None	738	15.65	12.81	18.48		None	770	16.57	13.83	19.30
**1993**	Degree	1356	18.18	15.56	20.79	**1994**	Degree	1308	17.61	15.39	19.82
	A-Level	2286	15.55	13.96	17.14		A-Level	2213	15.20	13.57	16.83
	GCSE	3508	15.90	14.61	17.18		GCSE	3445	14.83	13.55	16.11
	None	3424	17.34	15.98	18.70		None	3099	17.03	15.65	18.41
**1997**	Degree	892	12.23	9.97	14.49	**1998**	Degree	1642	14.66	12.75	16.56
	A-Level	1344	16.23	14.13	18.32		A-Level	2362	15.38	13.88	16.88
	GCSE	1687	15.01	13.21	16.80		GCSE	3243	15.23	13.90	16.56
	None	1496	16.69	14.69	18.70		None	2589	16.92	15.30	18.53
**1999**	Degree	868	15.62	13.07	18.17	**2000**	Degree	952	12.50	10.31	14.68
	A-Level	1193	15.32	13.20	17.45		A-Level	1382	10.75	9.08	12.42
	GCSE	1494	16.36	14.37	18.36		GCSE	1558	14.38	12.53	16.23
	None	1245	21.39	18.80	23.98		None	1152	19.12	16.45	21.80
**2001**	Degree	1881	12.40	10.82	13.99	**2002**	Degree	975	14.01	11.80	16.23
	A-Level	2501	12.87	11.54	14.20		A-Level	1299	15.52	13.45	17.59
	GCSE	3115	12.47	11.27	13.67		GCSE	1520	16.46	14.46	18.46
	None	2056	15.93	14.08	17.78		None	855	22.12	18.83	25.42
**2003**	Degree	1982	11.25	9.81	12.68	**2004**	Degree	943	13.10	10.86	15.35
	A-Level	2339	12.22	10.87	13.57		A-Level	1058	12.41	10.44	14.39
	GCSE	2940	12.98	11.74	14.22		GCSE	1224	10.94	9.15	12.73
	None	1797	17.16	15.05	19.27		None	884	18.26	15.36	21.17
**2005**	Degree	1094	10.98	9.08	12.87	**2006**	Degree	2217	10.51	9.19	11.83
	A-Level	1199	12.65	10.76	14.53		A-Level	2332	13.37	11.98	14.76
	GCSE	1389	13.56	11.75	15.37		GCSE	2536	13.73	12.36	15.11
	None	949	19.51	16.52	22.50		None	1606	19.69	17.47	21.91
**2008**	Degree	2388	11.04	9.76	12.32	**2009**	Degree	745	13.01	10.43	15.59
	A-Level	2604	12.55	11.28	13.83		A-Level	715	16.15	13.42	18.87
	GCSE	2669	14.82	13.44	16.20		GCSE	873	18.41	15.76	21.06
	None	1639	19.43	17.18	21.68		None	473	21.60	17.46	25.74
**2010**	Degree	1387	13.21	11.40	15.02	**2012**	Degree	1500	14.63	12.85	16.41
	A-Level	1375	15.43	13.51	17.35		A-Level	1314	14.17	12.30	16.05
	GCSE	1447	15.56	13.64	17.49		GCSE	1188	16.87	14.69	19.06
	None	673	21.54	17.86	25.22		None	648	19.35	16.07	22.63
**2014**	Degree	1533	12.96	11.25	14.67						
	A-Level	1273	13.61	11.68	15.53						

	GCSE	1145	15.13	13.00	17.26						
	None	581	23.71	20.04	27.38						

For peer review only

Appendix 7: Relative index of inequality in GHQ caseness, 1991-2014

(displayed in Figure 4 in main text)

Highest Education Level as Measure of SEP					Area-Level Deprivation as Measure of SEP				
Year	Incidence Rate Ratio	p-value	Lower 95% CI	Upper 95% CI	Year	Incidence Rate Ratio	p-value	Lower 95% CI	Upper 95% CI
**1991**	0.86	0.445	0.58	1.27					
**1992**	0.88	0.458	0.63	1.23					
**1993**	1.02	0.797	0.87	1.21					
**1994**	0.96	0.634	0.81	1.14					
**1997**	1.25	0.054	1.00	1.57					
**1998**	1.11	0.232	0.93	1.32					
**1999**	1.46	0.002	1.15	1.86					
**2000**	1.79	<0.001	1.37	2.33					
**2001**	1.26	0.018	1.04	1.53	**2001**	1.68	<0.001	1.40	2.02
**2002**	1.51	0.001	1.18	1.93	**2002**	1.93	<0.001	1.54	2.41
**2003**	1.57	<0.001	1.27	1.92	**2003**	1.95	<0.001	1.61	2.37
**2004**	1.35	0.066	0.98	1.86	**2004**	2.05	<0.001	1.52	2.77
**2005**	1.70	<0.001	1.29	2.24	**2005**	2.65	<0.001	2.03	3.47
**2006**	1.98	<0.001	1.61	2.43	**2006**	2.13	<0.001	1.74	2.60
**2008**	1.79	<0.001	1.47	2.18	**2008**	2.28	<0.001	1.89	2.76
**2009**	2.13	<0.001	1.52	2.99	**2009**	2.11	<0.001	1.54	2.91
**2010**	1.59	0.001	1.22	2.08	**2010**	1.85	<0.001	1.43	2.38
**2012**	1.46	0.005	1.12	1.90	**2012**	2.32	<0.001	1.80	2.99
**2014**	1.72	<0.001	1.31	2.26	**2014**	2.22	<0.001	1.72	2.87

For peer review only

BMJ Open

Trends in gender and socioeconomic inequalities in mental health following the Great Recession and subsequent austerity policies: a repeat cross-sectional analysis of the Health Surveys for England

Journal:	*BMJ Open*
Manuscript ID	bmjopen-2018-022924.R1
Article Type:	Research
Date Submitted by the Author:	05-Jun-2018
Complete List of Authors:	Thomson, Rachel; MRC/CSO Social & Public Health Sciences Unit, University of Glasgow; NHS Ayrshire and Arran, Public Health<br>Niedzwiedz, Claire; University of Glasgow Institute of Health and Wellbeing<br>Katikireddi, Srinivasa; MRC/CSO Social & Public Health Sciences Unit, University of Glasgow
**Primary Subject Heading**:	Mental health
Secondary Subject Heading:	Epidemiology, Public health, Sociology, Health policy
Keywords:	MENTAL HEALTH, PUBLIC HEALTH, SOCIAL MEDICINE, EPIDEMIOLOGY

SCHOLARONE™
Manuscripts

For peer review only

Trends in gender and socioeconomic inequalities in mental health following the Great Recession and subsequent austerity policies: a repeat cross-sectional analysis of the Health Surveys for England

Rachel M Thomson*[1, 2], Claire L Niedzwiedz[3], Srinivasa Vittal Katikireddi[1]

[1] MRC/CSO Social & Public Health Sciences Unit, University of Glasgow, UK

[2] Public Health Department, NHS Ayrshire & Arran, UK

[3] Institute of Health & Wellbeing, University of Glasgow, UK

*Corresponding author

Postal address:

Dr Rachel Thomson
Public Health Dept, Afton House
Ailsa Hospital Campus
Dalmellington Road
Ayr KA6 6AB

Email rachel.thomson8@nhs.net

Phone: 01292 885 858

Word count (excl. title page, abstract, references, figures and tables, declarations): 3315 words

For peer review only

ABSTRACT

Objective: It is known that mental health deteriorated following the 2008 global financial crisis, and that subsequent UK austerity policies post-2010 disproportionately impacted women and those in deprived areas. We aimed to assess whether gender and socioeconomic inequalities in poor mental health have changed since the onset of austerity policies.

Design: Repeat cross-sectional analysis of survey data.

Setting: England.

Participants: Nationally and regionally representative samples of the working age population (25-64 years) from the Health Survey for England (1991-2014).

Outcome Measures: Population-level poor mental health was measured by General Health Questionnaire-12 (GHQ) caseness, stratified by gender and socioeconomic position (area-level deprivation and highest educational attainment).

Results: The prevalence of age-adjusted male GHQ caseness increased by 5.9% (percentage points; 95% CI 3.2%-8.5%, P<0.001) from 2008 to 2009 in the immediate post-recession period, but recovered to pre-recession levels after 2010. In women, there was little change in 2009 or 2010, but an increase of 3.0% (95% CI 1.0%-5.1%, p=0.004) in 2012 compared to 2008 following the onset of austerity. Estimates were largely unchanged after further adjustment for socioeconomic position, employment status, and household income as potential mediators. Relative socioeconomic inequalities in GHQ caseness narrowed from 2008 to 2010 immediately following the recession, with relative index of inequality (RII) falling from 2.28 (95% CI 1.89-2.76, p<0.001) to 1.85 (95% CI 1.43-2.38, p<0.001), but returned to pre-recession levels during austerity.

Conclusions: Gender inequalities in poor mental health narrowed following the Great Recession but widened during austerity, creating the widest gender gap since 1994. Socioeconomic inequalities in poor mental health narrowed immediately post-recession, but this trend may now be reversing. Austerity policies could contribute to widening mental health inequalities.

Keywords: mental health; public health; social medicine; epidemiology

For peer review only

ARTICLE SUMMARY

Strengths and limitations of this study

- Data are from a large nationally and regionally representative survey, and our study considered trends over a long period of time using a validated measure of poor mental health.
- Inequalities in poor mental health were explored by both socioeconomic position (using two measures to demonstrate consistency of trends) and gender, rarely explicitly done in current literature.
- Lack of available data meant it was not possible to categorise individuals according to whether they were subject to specific austerity measures; further research with such data and a clear control group would strengthen arguments for causality.
- The use of cross-sectional rather than longitudinal data mean the ability to derive causal inferences is limited and further longitudinal work is required.

For peer review only

INTRODUCTION

The health and social repercussions of the 2008 Great Recession are still being felt today.[1, 2] Much existing research has focused on the relationship between the economic downturn, rises in unemployment, and worsening mental health outcomes.[3, 4] Mirroring historical trends, in the aftermath of the recession there was an improvement in all-cause mortality across Europe,[5] paradoxically accompanied by a sharp rise in suicide rates which disproportionately impacted men.[6]

There has been a growing call to interpret trends in mental health outcomes in the context of the political decisions that followed,[7, 8] particularly given that there was marked cross-national variation in these outcomes.[9] It has been argued that the pursuit of austerity policies in response to the recession, usually involving large-scale public sector reforms, may actually have worsened health outcomes and delayed economic recovery.[10-12] It has also been postulated that austerity policies may worsen inequalities in health outcomes, as they frequently result in cutbacks to programs aiming to address inequitable distribution of the social determinants of health such as housing and education.[13]

The package of austerity measures implemented by the UK Government in 2010 was the third largest in Europe, with substantial cuts especially to welfare, health and social care.[14] Between 2010 and 2015, £26 billion worth of cuts were made to benefits, tax credits, pay and pensions in the UK,[15] with local authorities serving more deprived communities seeing greater financial losses.[16] 85% of financial savings from welfare reforms have been taken from the incomes of women, largely due to the fact that they make up the majority of lone parents and unpaid carers.[17] Women also form a large proportion of the public sector workforce, two-thirds in 2012-13,[18] so are more likely to have been impacted by the two year public sector pay freeze in 2010 and subsequent 1% pay cap that has led to a pay cut in real terms.[14]

Our previous research demonstrated an increase in poor mental health in men but not women following the Great Recession, with no clear evidence for an increase in socioeconomic inequalities.[19]

We aimed for the first time to investigate trends in both gender inequalities and socioeconomic inequalities in poor mental health in the UK following the onset of austerity, and compare these to the immediate aftermath of the 2008 recession.

For peer review only

METHODS

Dataset

Following our previous approach, we used the Health Survey for England (HSE; 1991-2014), a multi-stage stratified random sample designed to be nationally and regionally representative, to construct a repeat cross-sectional dataset. Details of the HSE have been published elsewhere.[20] Response levels have fallen over time but plateaued recently, remaining reasonably high at 62% in 2014 compared with 64% in 2007.[21] Weights for non-response were available from 2003. The rationale for choosing this dataset was the lengthy time period over which it has run using standardised methods, allowing consideration of very long-term trends.

Population

The HSE general population samples were used for all analyses, restricted to those between 25 and 64 years of age to minimise misclassification of employment status among students. Those missing data on age, gender, measure of socioeconomic position (SEP), employment status or outcome were excluded. Sensitivity analysis was performed using the population aged 25-59 years to ensure inclusion of early retirees was not impacting results.

From 1991-2014 there were 128,003 potential participants. 7,774 participants (6.1%) missing outcome data, 109 (0.1%) missing educational attainment, 2964 (2.3%) with foreign or other qualifications which could not be categorised, and 37 (0.03%) missing employment status were excluded, leaving 117,119 participants (91.5%) for inclusion. For analysis using area-level deprivation from 2001 onwards where there were 73,682 potential participants, 5,317 participants (7.2%) missing outcome data, 562 participants (0.7%) missing deprivation score, and 25 (0.03%) missing employment status were excluded, leaving 67,778 participants for inclusion (92.0%).

Exposure Measurement and Covariates

The SEP exposure measures considered were educational attainment and area-level deprivation. Highest educational attainment was available for all years except 1995 and 1996, coded into four categories: degree-level or equivalent, A-level or equivalent, GCSE or equivalent, and no formal qualifications. A marker of small area-level deprivation based on postcode (Index of Multiple Deprivation [IMD] scored in quintiles) was available from 2001.

Covariates considered were employment status and total household income. Employment status was recorded as self-reported activity within the preceding week, coded in six categories: in

For peer review only

employment, unemployed, retired through ill health, retired, looking after home, or in education. Total household income was available from 1997, coded into quintiles.

The UK economy did not enter recession until the last quarter of 2008 (defined by two successive quarters of negative growth in GDP),[22, 23] and while austerity policies were announced in mid-2010[14] it is unlikely that health consequences would have manifested within this year. We therefore defined in advance all years up to and including 2008 'pre-recession', the years 2009 and 2010 the 'recession period' and from 2012 onwards the 'austerity period' (outcome data were unavailable for 2011).

Outcome Measurement

Poor mental health was assessed using the General Health Questionnaire-12 (GHQ-12), a validated screening tool for common mental health problems used widely in epidemiological research, which scores self-reported symptoms of anxiety and depression.[24] The GHQ-12 formed part of the core questions in each sweep of the HSE except 1996 and 2007, though from 2010 has only been included every second year. A GHQ-12 score of four or greater indicates a strong likelihood of a common mental disorder,[25] and therefore defined a 'case'.

Statistical Analysis

Directly age-standardised prevalence estimates of GHQ caseness were calculated for each year, stratified by gender and both measures of SEP. The 2013 WHO European Standard Population was used for all direct standardisations, and estimates were displayed graphically.

To quantify any potential impact of the recession and austerity on mental health by gender, multivariable logistic regression modelling was performed. First, data from each year were regressed separately to determine long-term trends in the difference between male and female caseness, adjusting for age, education, and employment status. In a combined dataset of all years, models for men and women separately were then created using 2008 as the baseline/pre-recession year, and adjusted for age, SEP, employment status, and total household income. As the main time period of interest was following the point at which IMD was recorded routinely, we focused on this as the primary measure of SEP, given marked changes in the distribution of educational attainment over the study period. In addition to odds ratios, adjusted prevalence differences were derived from the logistic regression models to give a measure of change on the absolute scale.

Long-term trends in socioeconomic inequalities in mental health over time were analysed using the relative index of inequality (RII), a regression-based index comparing the prevalence of the outcome between those of the theoretically lowest and highest SEP, thus giving a relative measure that could

For peer review only

be used to draw comparisons irrespective of changes in group composition over time.[26] Analysis was performed using both SEP measures. Participants were ranked according to the chosen measure of SEP within the datasets for each individual year, with tied participants receiving the same rank; these ranks were then divided by the sample size, scaling the rank value to between 0 and 1 with a mean of 0.5.[27] Poisson regression was used to generate prevalence risk ratios with 95% confidence intervals,[28] comparing the most deprived with the least deprived group, which were then plotted to view trends. All models were adjusted for age and sex.

Patient and Public Involvement

There was no patient or public involvement in the design of this study.

For peer review only

RESULTS

Characteristics of included individuals are displayed in supplementary appendix 1. Over the study period there was a marked increase in women reporting degree-level education, and for both genders the number reporting no formal qualifications fell. During the main time period of interest (2005 onwards) there was little change in gender distribution.

Mental Health Trends by Gender

The prevalence of GHQ caseness was consistently higher in women than men over the study period (Figure 1). There were three clear points of deviation from secular trends for both genders: the late 1990s, early 2000s, and 2008 onwards. These deviations coincide with periods of macroeconomic disruption. During the former two time periods the UK economy declined but avoided entering recession;[22, 23] the increases in prevalence which coincide with these were patterned similarly between genders. Conversely, in 2009 following the Great Recession there was a marked increase in age-standardised GHQ caseness in men and a more modest increase in women, with only a slight improvement for men in 2010. During the austerity period this patterning altered. While in 2012 male GHQ caseness continued to decrease, female GHQ caseness increased to 18.7% (95% CI 17.2-20.2), its highest observed value since 2002.

[INSERT FIGURE 1]

Between 1991 and 2004 the difference between male and female prevalence, adjusted for age, education and employment status, narrowed from 7.9% (95% CI 4.3-11.4) to 3.3% (95% CI 1.1-5.6). Despite a marked further narrowing of this gender gap in the recession period with a fall to 1.1% (95% CI -1.8-4.0) in 2009 (secondary to the more marked increase in GHQ caseness for men), by 2012 it had sharply widened again to 6.8% (95% CI 4.6-8.9), the largest adjusted difference between male and female prevalence since 1994. Values for all years are provided in supplementary appendices 2 and 3.

Table 1 presents logistic regression models from the period of interest for each gender, with tables for the whole time period 2001-2014 available in supplementary appendix 4.

For peer review only

Table 1: Multiple logistic regression models (with odds ratios and % point difference) for participants of each gender, 2005-2014 (2008 as pre-recession reference year)

Regression models for men (n=24,930)												
	Model 1: Adjusted for age, IMD				Model 2: Adjusted for age, IMD, employment				Model 3: Adjusted for age, IMD, employment, income			
**Year**	**Odds Ratio**	**p-value**	**Lower 95% CI**	**Upper 95% CI**	**Odds Ratio**	**p-value**	**Lower 95% CI**	**Upper 95% CI**	**Odds Ratio**	**p-value**	**Lower 95% CI**	**Upper 95% CI**
2005	1.03	0.722	0.86	1.24	0.92	0.400	0.76	1.12	0.90	0.303	0.74	1.10
2006	1.06	0.476	0.91	1.24	1.04	0.676	0.88	1.22	1.03	0.765	0.87	1.21
2008	1.00	-	-	-	1.00	-	-	-	1.00	-	-	-
2009	1.64	<0.001	1.34	2.00	1.55	<0.001	1.25	1.93	1.53	<0.001	1.24	1.91
2010	1.28	0.009	1.06	1.53	1.26	0.021	1.04	1.52	1.26	0.018	1.04	1.53
2012	1.15	0.147	0.95	1.38	1.10	0.340	0.91	1.33	1.10	0.342	0.91	1.33
2014	1.13	0.215	0.93	1.37	1.17	0.126	0.96	1.43	1.18	0.108	0.96	1.44
**Year**	**% Diff.**	**p-value**	**Lower 95% CI**	**Upper 95% CI**	**% Diff.**	**p-value**	**Lower 95% CI**	**Upper 95% CI**	**% Diff.**	**p-value**	**Lower 95% CI**	**Upper 95% CI**
2005	0.34	0.723	-1.53	2.20	-0.75	0.396	-2.50	0.99	-0.93	0.298	-2.67	0.82
2006	0.58	0.477	-1.01	2.17	0.33	0.676	-1.21	1.86	0.23	0.765	-1.30	1.77
2008	0.00	-	-	-	0.00	-	-	-	0.00	-	-	-
2009	5.88	<0.001	3.24	8.52	4.72	<0.001	2.20	7.23	4.62	<0.001	2.11	7.12
2010	2.67	0.012	0.60	4.73	2.29	0.024	0.30	4.28	2.37	0.021	0.36	4.38
2012	1.44	0.154	-0.54	3.42	0.89	0.344	-0.95	2.73	0.89	0.346	-0.96	2.74
2014	1.26	0.223	-0.77	3.30	1.53	0.134	-0.47	3.52	1.63	0.115	-0.40	3.65
**Regression models for women (n=31,413)**												
	Model 1: Adjusted for age, IMD				Model 2: Adjusted for age, IMD, employment				Model 3: Adjusted for age, IMD, employment, income			
**Year**	**Odds Ratio**	**p-value**	**Lower 95% CI**	**Upper 95% CI**	**Odds Ratio**	**p-value**	**Lower 95% CI**	**Upper 95% CI**	**Odds Ratio**	**p-value**	**Lower 95% CI**	**Upper 95% CI**
2005	1.06	0.426	0.92	1.22	1.05	0.531	0.91	1.21	1.02	0.762	0.88	1.18
2006	0.91	0.153	0.81	1.03	0.90	0.102	0.80	1.02	0.89	0.077	0.79	1.01
2008	1.00	-	-	-	1.00	-	-	-	1.00	-	-	-
2009	1.06	0.537	0.89	1.25	1.07	0.429	0.90	1.28	1.07	0.431	0.90	1.28
2010	1.00	0.980	0.86	1.15	0.99	0.849	0.85	1.14	0.99	0.922	0.86	1.15
2012	1.24	0.003	1.08	1.42	1.24	0.003	1.07	1.43	1.25	0.002	1.09	1.45
2014	1.10	0.208	0.95	1.27	1.11	0.171	0.96	1.28	1.12	0.138	0.97	1.29
**Year**	**% Diff.**	**p-value**	**Lower 95% CI**	**Upper 95% CI**	**% Diff.**	**p-value**	**Lower 95% CI**	**Upper 95% CI**	**% Diff.**	**p-value**	**Lower 95% CI**	**Upper 95% CI**
2005	0.79	0.429	-1.16	2.74	0.60	0.533	-1.29	2.50	0.29	0.762	-1.60	2.18
2006	-1.16	0.153	-2.74	0.43	-1.28	0.102	-2.82	0.25	-1.39	0.077	-2.94	0.15
2008	0.00	-	-	-	0.00	-	-	-	0.00	-	-	-
2009	0.73	0.541	-1.61	3.07	0.93	0.435	-1.40	3.25	0.93	0.436	-1.41	3.27
2010	-0.02	0.98	-1.96	1.91	-0.18	0.848	-2.07	1.70	-0.10	0.922	-2.00	1.81

For peer review only

2012	3.04	0.004	0.99	5.08	2.90	0.004	0.93	4.88	3.11	0.002	1.11	5.11
2014	1.27	0.213	-0.73	3.28	1.34	0.176	-0.60	3.29	1.47	0.142	-0.49	3.44

GHQ caseness in men was higher in both 2009 and 2010 after adjusting for age and IMD, with prevalence predicted to have increased in the population by 5.9% (95% CI 3.2-8.5, p<0.001) percentage points from 2008 to 2009. This increase remained largely unchanged (4.6%, 95% CI 2.1-7.1, p<0.001) after adjustment for the potential mediating effect of employment status and household income. For men, there was no evidence of significant worsening of population mental health in either 2012 or 2014 when compared with 2008 in any model.

For women, after adjusting for age and IMD there was no evidence of an increase in GHQ caseness during the recession period. However, in 2012 the predicted increase in the population compared with 2008 was 3.0% (95% CI 1.0-5.1, p=0.004), and after further adjustment for employment status and household income this remained largely unchanged at 3.1% (95% CI 1.1-5.1, p=0.002). There was a smaller adjusted increase in 2014 compared with 2008 of 1.5% (95% CI -0.5-3.4, p=0.142).

Mental Health Trends by Socioeconomic Position

[INSERT FIGURE 2]

There was a clear socioeconomic gradient in GHQ caseness throughout the study period (Figure 2). The absolute difference between the most and least deprived quintiles was amongst the highest recorded during the austerity period (13.5% in 2012, 11.2% in 2014) compared with smaller differences during the recession period (9.2% in 2009, 8.6% in 2010). All values are provided in supplementary appendix 5.

[INSERT FIGURE 3]

Stratification by highest educational attainment produced similar trends during the recession and austerity periods (Figure 3), with the exception of those with no formal qualifications. This group experienced worsening of GHQ caseness throughout the study period, rising from 15.7% (95% CI 12.8-18.5) in 1991 to 23.7% (95% CI 20.0-27.4) by 2014 without seeing the recovery experienced by other groups during the austerity period. All values are provided in supplementary appendix 6. To explore the marked worsening for the least educated during the austerity period, further stratification by gender was performed for the period 2012-2014: the increase in this group was predominantly among men, with age-standardised prevalence rising from 16.0% (95% CI 11.5-20.5) in 2012 to 22.8% (95% CI 17.3-28.3) in 2014, while for women the increase was smaller from 22.7% (95% CI 17.9-27.5) in 2012 to 24.7% (95% CI 19.8-29.5) in 2014.

For peer review only

[INSERT FIGURE 4]

Relative socioeconomic inequalities in GHQ caseness have been consistently observed since 1999 (Figure 4). Inequalities in GHQ caseness have increased from the late 1990s to the immediate pre-recession period, with inequalities generally larger by area-level deprivation. During the recession period there was a slight reduction in socioeconomic inequalities, with RII by education falling from 1.8 (95% CI 1.5-2.2, p<0.001) in 2008 to 1.6 (95% CI 1.2-2.1, p=0.001) in 2010 and by IMD quintile from 2.3 (95% CI 1.9-2.8, p<0.001) in 2008 to 1.9 (95% CI 1.4-2.4, p<0.001) in 2010. However, these trends reversed during the austerity period, and by 2014 both RIIs had returned to pre-recession levels. All values are provided in supplementary appendix 7.

For all analyses, sensitivity analysis excluding those aged 60-64 years did not affect trends.

For peer review only

DISCUSSION

In this large repeat cross-sectional study of a representative sample of the English population, we found mental health worsened for women following the onset of austerity policies, while men saw a recovery to pre-recession levels. As a result of the changes, gender inequalities in poor mental health widened during the austerity period, reversing the trend from 1991-2004 of gradual improvement. We also found that socioeconomic inequalities in poor mental health narrowed in the immediate years following the 2008 recession but widened during the austerity period. While it is not possible to draw definitive causal conclusions from this study, our findings are useful in examining changes in secular trends and their chronological association with macroeconomic events and policies.

There is conflicting evidence in existing literature around whether mental health inequalities by gender or socioeconomic position have widened in the UK since the recession. Our previous work suggested males saw the sharpest worsening of mental health, and found no evidence of widening socioeconomic inequalities when existing trends were taken into account.[19] However, this was prior to the onset of austerity. More recent evidence showed a more marked worsening of mental health for women in 2014 compared with 2007, but did not take into account intervening years.[29] A large study of pan-European data including the UK found no systematic influence of the recession on socioeconomic inequalities in depression up to 2014,[30] but did not differentiate between the immediate recessionary period and the period following any economic policy response. Work by Barr et al suggested that from 2009 to 2013 there may have been a widening of socioeconomic inequalities in mental health in the UK.[31] However, this used self-reported diagnoses and only two broad categories of socioeconomic group. Our study adds clarity to both areas.

There is no consensus around what factors are responsible for the gender gap in poor mental health. There is little evidence it results from purely genetic or biological differences, with sociocultural roles, adverse life events and learned psychological attributes thought more likely contributing factors.[32] Our findings of a reversal in trend direction echo those of others who have begun to raise concerns about the mental health of UK women in recent years, particularly young women.[29, 33] The timing of this reversal in relation to austerity reforms, and the differential gender patterning of austerity,[17] could indicate that the change for women may be secondary to the policy response rather than the economic crisis itself – particularly, as evidence emerges of likely adverse impacts of specific policy reforms affecting women, such as restrictions to income support being linked to deteriorations in mental health amongst lone parents.[34]

For peer review only

The finding of a reversal in trend towards widening socioeconomic inequalities following the onset of austerity adds to the evidence base arguing such measures may mediate the link between macroeconomic change and mental health.[2, 10] Ecological studies using pan-European data suggest the direct effect of unemployment on suicide rates was greater in countries with lower social spending,[35] and conversely, higher government spending on unemployment support may mitigate adverse impacts on self-rated health.[36] On a relative scale the widening of socioeconomic inequalities post-austerity is small in the context of long-term trends, particularly by highest qualification (Figure 4), and the degree and timing of short-term trends around the recession and austerity period also differ between measures of SEP, possibly explaining the current lack of consensus in the literature.[30, 31]

The marked divergence for those with no formal qualifications by 2014 may support the hypothesis that those in low-skilled jobs (who are known to experience poorer health outcomes[37]) may be worst affected by reduced in-work financial support or worsening job conditions such as increased insecure work.[38] Their divergence may also be partly attributable to changes in demographics over the study period, with the group achieving no qualifications becoming smaller and more homogenous over time. Regardless, they are notable outliers in 2014, identifying this group as particularly high risk for poor mental health.

Strengths and Limitations

Our study has a number of important strengths. The HSE is a large, nationally and regionally representative survey which has used standard protocols over a long period of time. The GHQ-12 is a validated and commonly used measure, and outcome data were available for most years allowing detailed consideration of trends. While there is some debate about the most appropriate threshold to use to determine caseness in different populations,[39] we chose a cut-off value that has been used previously with this population[19, 40] and which indicates a strong likelihood of common mental disorder,[25] increasing specificity and reducing the likelihood of false positive cases. The use and comparison of two measures of SEP is useful in demonstrating consistency of trends between SEP and poor mental health.

Our study also has some limitations which must be considered. The use of cross-sectional rather than individual longitudinal data mean the ability to derive causal inferences is limited; however, it does overcome attrition bias in cohort studies which can commonly lead to an underestimation of inequalities.[41] As data were not collected on whether individuals were subject to specific austerity measures, this could not be included as an explanatory variable. Household income was felt to be a reasonable proxy given that most reforms were associated with financial loss.[16] It is acknowledged

For peer review only

that the impact of an economic crisis or subsequent policies is not necessarily immediate and is likely to be mediated by related factors such as long-term unemployment. It is therefore possible that trends in GHQ caseness may have been influenced by other factors apart from austerity, including observed trends reflecting the longer term impacts of earlier macro-economic exposures. Finally, it is unfortunate that outcome data were not available from 2007, 2011, 2013 or 2015, as this would have strengthened the evidence for the assessment of trends.

Further research using longitudinal data would add strength to any argument for causality, as would replication using alternative outcome measures, such as antidepressant prescriptions. Distinguishing between the impact of different components of austerity measures, e.g. public sector employment terms, welfare reforms, or access to community services, could add further nuance to our reporting of their potential combined impact and overcome this identified limitation. Furthermore, increasing devolution provides the opportunity to study differences in policy approaches within the UK.[42] Cross-national comparisons would also be useful in determining whether observed trends are replicated elsewhere, and whether impacts are dependent on levels of austerity, and natural experiment approaches could strengthen causal inference.[43] Finally, it is clearly important to see whether the observed trajectories in mental health inequalities have continued following 2014, particularly given that more severe welfare reforms were initiated in 2015.[16]

Conclusions

This study adds to what the European Psychiatric Association in 2016 described as an emerging 'broad consensus about the deleterious consequences of economic crises on mental health'.[44] The gender gap in mental health, which had been improving prior to the recession, appears to be sharply widening again following the onset of austerity policies which have largely focused on women. Those in the most deprived groups have been shown to be at potentially heightened risk of poor mental health following the onset of austerity, with the least educated at highest risk.

These findings are alarming, particularly given that since the time period studied there have been further cuts to mental health provision which mean the issue may now be worse.[45] Labonté and Stuckler argue in strong terms that, based on current evidence of economic, health and social harms, austerity policies threaten to 'imperil the world's population' without radical reform.[2] Policymakers in the UK, and those considering embarking on or continuing austerity measures elsewhere in the world, should be aware that these may have adverse health impacts for their populations.

For peer review only

DECLARATIONS

Acknowledgements

We would like to thank Frank Popham for providing feedback on drafts of the manuscript. We are extremely grateful to the individuals who took part in all cycles of the Health Survey for England, and all those involved in its administration over the years. We would like to acknowledge the assistance of the UK Data Archive for providing access to the data; the Information Centre for health and social care and Department of Health for sponsoring the Health Survey for England; and the Principal Investigators of the Health Survey for England, Natcen Social Research and the Department of Epidemiology and Public Health at the Royal Free and University College Medical School. The responsibility for the analysis presented here lies solely with the authors.

Competing Interests

The authors declare no conflicts of interest.

Funding

SVK is funded by a NRS Senior Clinical Fellowship (SCAF/15/02), the Medical Research Council (MC_UU_12017/13 & MC_UU_12017/15) and Scottish Government Chief Scientist Office (SPHSU13 & SPHSU15). CLN is funded by the Medical Research Council (MR/R024774/1).

Contributors

RT serves as guarantor for this article. RT and SVK conceived the idea for the study, and RT performed the statistical analysis and wrote the first draft of the article. CN and SVK assisted in research design, interpretation of findings and critical revision of the manuscript.

Ethics Approval

Ethical approval was not required for this study as it used previously collected data. Ethical approval for each year of the survey was obtained by the Health Survey for England team.

Data Sharing Statement

No additional data available.

Figure Legends

Figure 1: Age-standardised General Health Questionnaire caseness in men and women aged 25-64 years, 1991-2014, with 95% confidence intervals; Percentage point difference between male and

For peer review only

female GHQ caseness with 95% confidence intervals, adjusted for age, education and employment status using logistic regression. Dashed lines indicate missing years.

Figure 2: Age-sex standardised General Health Questionnaire caseness by Index of Multiple Deprivation quintile in 25-64 year olds, 2001-2014, with 95% confidence intervals. Dashed lines indicate missing years.

Figure 3: Age-sex standardised General Health Questionnaire caseness by education level in 25-64 year olds, 1991-2014, with 95% confidence intervals. Dashed lines indicate missing years.

Figure 4: Relative index of inequality in General Health Questionnaire caseness in 25-64 year olds by education level and Index of Multiple Deprivation quintile, 1991-2014, with 95% confidence intervals. Dashed lines indicate missing years.

For peer review only

REFERENCES

1. Stuckler D, Basu S, Suhrcke M, et al. The public health effect of economic crises and alternative policy responses in Europe: an empirical analysis. *Lancet* 2009;374(9686):315-23. doi: 10.1016/S0140-6736(09)61124-7

2. Labonte R, Stuckler D. The rise of neoliberalism: how bad economics imperils health and what to do about it. *J Epidemiol Community Health* 2016;70(3):312-18. doi: 10.1136/jech-2015-206295

3. Frasquilho D, Matos MG, Salonna F, et al. Mental health outcomes in times of economic recession: a systematic literature review. *BMC Public Health* 2016;16:115. doi: 10.1186/s12889-016-2720-y [published Online First: 2016/02/06]

4. Katikireddi SV, Niedzwiedz CL, Popham F. Employment status and income as potential mediators of educational inequalities in population mental health. *Eur J Public Health* 2016;26(5):814-16. doi: 10.1093/eurpub/ckw126

5. Tapia Granados JA, Ionides EL. Population health and the economy: Mortality and the Great Recession in Europe. *Health Econ* 2017 doi: 10.1002/hec.3495 [published Online First: 2017/03/28]

6. Toffolutti V, Suhrcke M. Assessing the short term health impact of the Great Recession in the European Union: a cross-country panel analysis. *Prev Med* 2014;64:54-62. doi: 10.1016/j.ypmed.2014.03.028 [published Online First: 2014/04/11]

7. Karanikolos M, Heino P, McKee M, et al. Effects of the Global Financial Crisis on Health in High-Income Oecd Countries: A Narrative Review. *Int J Health Serv* 2016;46(2):208-40. doi: 10.1177/0020731416637160

8. Stuckler D, Basu S. The Body Economic: Eight experiments in economic recovery, from Iceland to Greece. London: Penguin 2013.

9. Reeves A, McKee M, Stuckler D. Economic suicides in the Great Recession in Europe and North America. *Br J Psychiatry* 2014;205(3):246-7. doi: 10.1192/bjp.bp.114.144766 [published Online First: 2014/06/14]

10. Karanikolos M, Mladovsky P, Cylus J, et al. Financial crisis, austerity, and health in Europe. *Lancet* 2013;381(9874):1323-31. doi: 10.1016/s0140-6736(13)60102-6 [published Online First: 2013/04/02]

11. De Vogli R. The financial crisis, health and health inequities in Europe: the need for regulations, redistribution and social protection. *Int J Equity Health* 2014;13:58. doi: 10.1186/s12939-014-0058-6 [published Online First: 2014/07/26]

For peer review only

12. Loopstra R, McKee M, Katikireddi SV, et al. Austerity and old-age mortality in England: a longitudinal cross-local area analysis, 2007-2013. *J R Soc Med* 2016;109(3):109-16. doi: 10.1177/0141076816632215

13. Ruckert A, Labonte R. The global financial crisis and health equity: early experiences from Canada. *Global Health* 2014;10:2. doi: 10.1186/1744-8603-10-2 [published Online First: 2014/01/08]

14. Reeves A, Basu S, McKee M, et al. Austere or not? UK coalition government budgets and health inequalities. *J R Soc Med* 2013;106(11):432-6. doi: 10.1177/0141076813501101

15. Engender, Scottish Refugee Council, Scottish Women's Aid, et al. A Widening Gap: Women and Welfare Reform. Scotland: Engender, 2015.

16. Beatty C, Fothergill S. The uneven impact of welfare reform: The financial losses to places and people. Sheffield: Centre for Regional Economic and Social Research, Sheffield Hallam University, 2016.

17. Ariss A, Firmin C, Meacher B, et al. Where's the Benefit? An Independent Inquiry into Women and Jobseeker's Allowance. In: Society TF, ed. London: The Fawcett Society, 2015.

18. Cribb J, Disney R, Sibieta L. The public sector workforce: past, present and future. London, 2014.

19. Katikireddi SV, Niedzwiedz CL, Popham F. Trends in population mental health before and after the 2008 recession: a repeat cross-sectional analysis of the 1991-2010 Health Surveys of England. *BMJ Open* 2012;2(5) doi: 10.1136/bmjopen-2012-001790

20. Mindell J, Biddulph JP, Hirani V, et al. Cohort profile: the health survey for England. *Int J Epidemiol* 2012;41(6):1585-93. doi: 10.1093/ije/dyr199 [published Online First: 2012/01/19]

21. NatCen Social Research, University College London, Department of Epidemiology and Public Health. Health Survey for England: UK Data Service, 1991-2014.

22. Macrotrends LLC. FTSE 100 Index - 32 Year Historical Chart [Online]: Macrotrends LLC; [updated June 2017; cited 23rd June 2017]. Available from: http://www.macrotrends.net/2598/ftse-100-index-historical-chart-data.

23. The Office for National Statistics. Times Series: Gross Domestic Product: Quarter on Quarter Growth: CVM SA%: ONS; [updated May 2017; cited 30th June 2017]. Available from: https://www.ons.gov.uk/economy/grossdomesticproductgdp/timeseries/ihyq/pn2.

24. Goldberg DP, Gater R, Sartorius N, et al. The validity of two versions of the GHQ in the WHO study of mental illness in general health care. *Psychol Med* 1997;27(1):191-7. [published Online First: 1997/01/01]

For peer review only

25. Goldberg DP, Williams, P. A user's guide to the General Health Questionnaire. Windsor: NFER-Nelson 1988.

26. Sergeant JC, Firth D. Relative index of inequality: definition, estimation, and inference. *Biostatistics* 2006;7(2):213-24. doi: 10.1093/biostatistics/kxj002 [published Online First: 2005/09/30]

27. Batty GD, Der G, Macintyre S, et al. Does IQ explain socioeconomic inequalities in health? Evidence from a population based cohort study in the west of Scotland. *The BMJ* 2006;332(7541):580-4. doi: 10.1136/bmj.38723.660637.AE [published Online First: 2006/02/03]

28. Mood C. Logistic Regression: Why We Cannot Do What We Think We Can Do, and What We Can Do About It. *Eur Sociol Rev* 2010;26(1):67-82.

29. McManus S, Bebbington P, Jenkins R, et al. Mental health and wellbeing in England: Adult Psychiatric Morbidity Survey 2014. Leeds: NHS Digital 2016.

30. Reibling N, Beckfield J, Huijts T, et al. Depressed during the depression: has the economic crisis affected mental health inequalities in Europe? Findings from the European Social Survey (2014) special module on the determinants of health. *Eur J Public Health* 2017;27(suppl_1):47-54. doi: 10.1093/eurpub/ckw225 [published Online First: 2017/03/30]

31. Barr B, Kinderman P, Whitehead M. Trends in mental health inequalities in England during a period of recession, austerity and welfare reform 2004 to 2013. *Soc Sci Med* 2015;147:324-31. doi: 10.1016/j.socscimed.2015.11.009

32. Piccinelli M, Wilkinson G. Gender differences in depression. Critical review. *Br J Psychiatry* 2000;177:486-92. [published Online First: 2000/12/05]

33. Lessof C, Ross A, Brind R, et al. Longitudinal Study of Young People in England cohort 2: health and wellbeing at wave 2; Research report UK: TNS BMRB, 2016.

34. Katikireddi SV, Molaodi OR, Gibson M, et al. Evaluating the impacts of restrictions to income support for lone parents on health: A natural experiment study. *The Lancet Public Health [in press]* 2018

35. Baumbach A, Gulis G. Impact of financial crisis on selected health outcomes in Europe. *Eur J Public Health* 2014;24(3):399-403. doi: 10.1093/eurpub/cku042 [published Online First: 2014/04/09]

36. Toge AG. Health Effects of Unemployment in Europe During the Great Recession: The Impact of Unemployment Generosity. *Int J Health Serv* 2016;46(4):614-41. doi: 10.1177/0020731416664688 [published Online First: 2016/08/27]

For peer review only

37. Katikireddi SV, Leyland AH, McKee M, et al. Patterns of mortality by occupation in the UK, 1991-2011: a comparative analysis of linked census and mortality records. *Lancet Public Health* 2017;2(11):e501-e12. doi: 10.1016/S2468-2667(17)30193-7

38. Benach J, Vives A, Amable M, et al. Precarious employment: understanding an emerging social determinant of health. *Annu Rev Public Health* 2014;35:229-53. doi: 10.1146/annurev-publhealth-032013-182500

39. Goldberg DP, Oldehinkel T, Ormel J. Why GHQ threshold varies from one place to another. *Psychol Med* 1998;28(4):915-21.

40. Maheswaran H, Kupek E, Petrou S. Self-reported health and socio-economic inequalities in England, 1996-2009: Repeated national cross-sectional study. *Soc Sci Med* 2015;136-137:135-46. doi: 10.1016/j.socscimed.2015.05.026 [published Online First: 2015/05/26]

41. Howe LD, Tilling K, Galobardes B, et al. Loss to follow-up in cohort studies: bias in estimates of socioeconomic inequalities. *Epidemiology* 2013;24(1):1-9. doi: 10.1097/EDE.0b013e31827623b1 [published Online First: 2012/12/06]

42. Katikireddi SV, Smith KE, Stuckler D, et al. Devolution of power, revolution in public health? *J Public Health (Oxf)* 2017;39(2):241-47. doi: 10.1093/pubmed/fdw031

43. Craig P, Katikireddi SV, Leyland A, et al. Natural Experiments: An Overview of Methods, Approaches, and Contributions to Public Health Intervention Research. *Annu Rev Public Health* 2017;38:39-56. doi: 10.1146/annurev-publhealth-031816-044327

44. Martin-Carrasco M, Evans-Lacko S, Dom G, et al. EPA guidance on mental health and economic crises in Europe. *Eur Arch Psychiatry Clin Neurosci* 2016;266(2):89-124. doi: 10.1007/s00406-016-0681-x [published Online First: 2016/02/15]

45. British Medical Association. Mental health budgets cut [Online] [updated February 2017; cited October 2017]. Available from: https://www.bma.org.uk/news/2017/february/mental-health-budgets-cut.

For peer review only

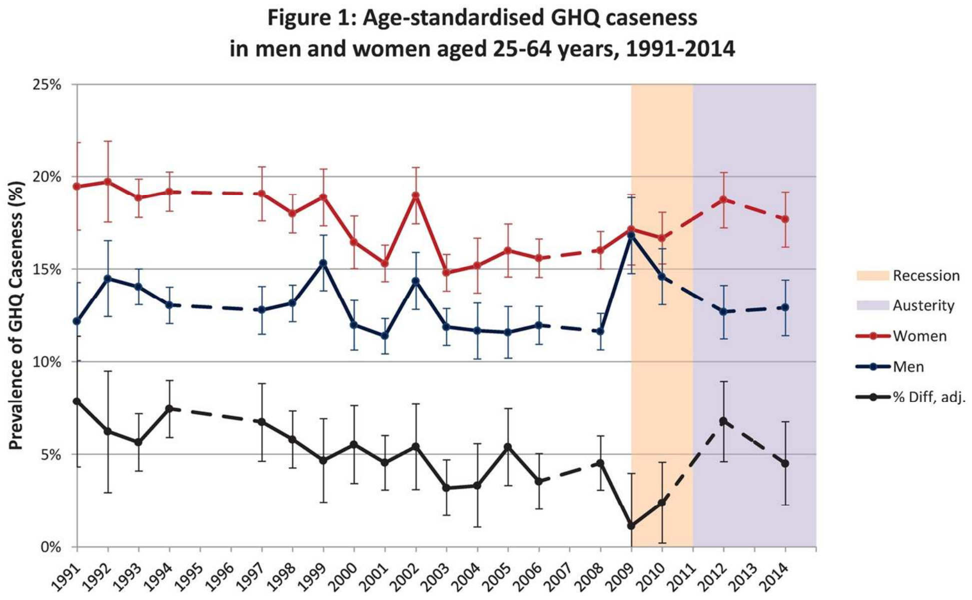

130x80mm (300 x 300 DPI)

Figure 2: Age-sex standardised GHQ caseness by IMD quintile in 25-64 year olds, 2001-2014

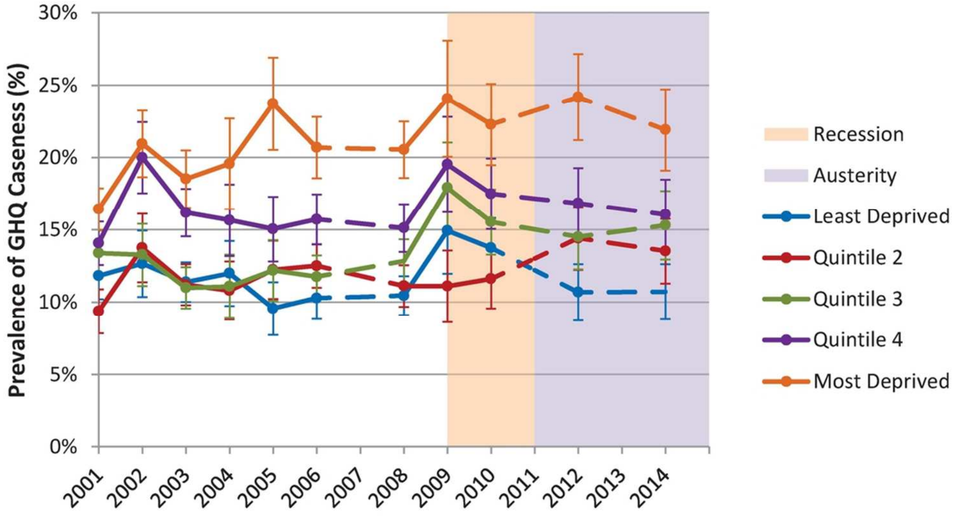

99x63mm (300 x 300 DPI)

view only

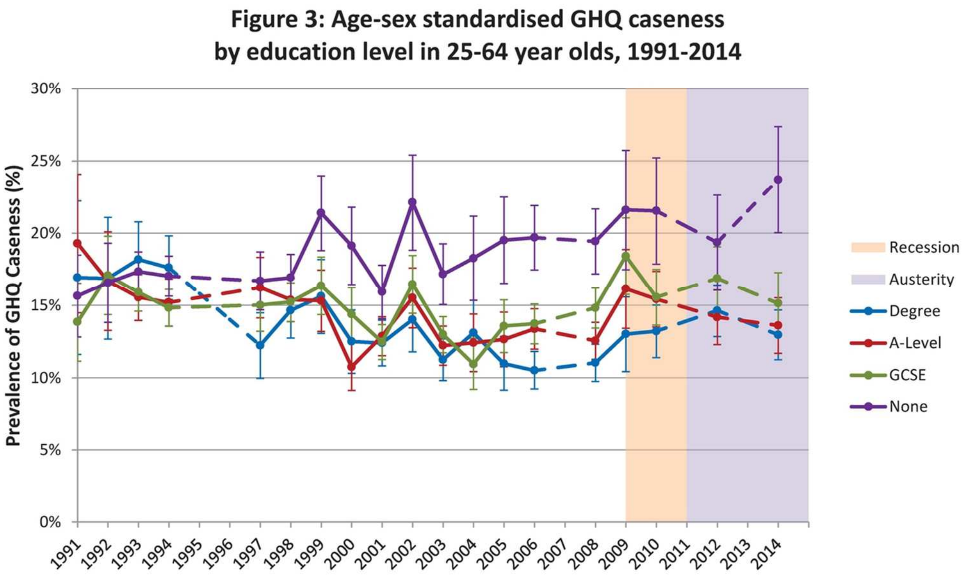

119x71mm (300 x 300 DPI)

For peer review only

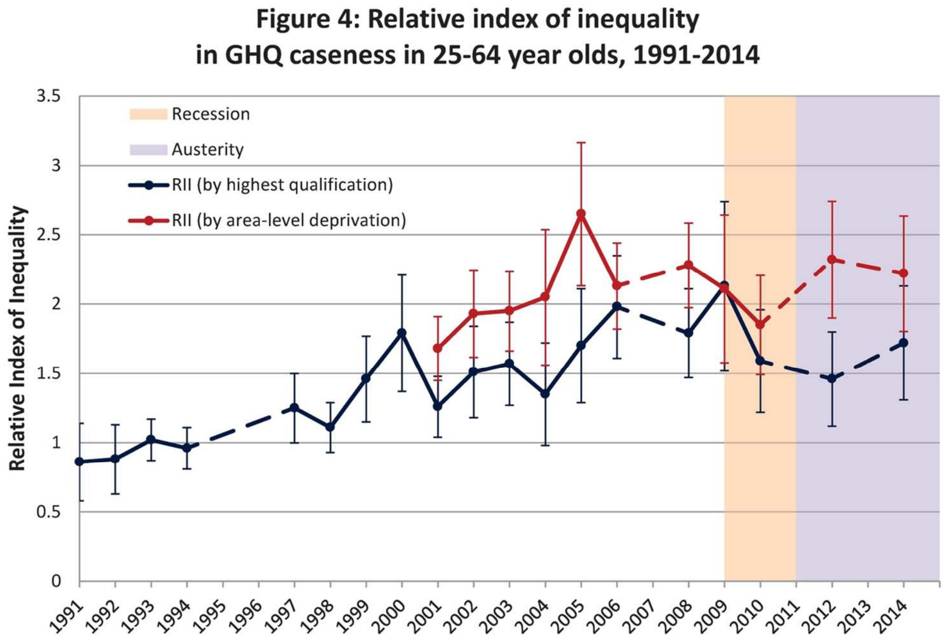

119x79mm (300 x 300 DPI)

For peer review only

Supplementary Appendix

Appendix 1: Characteristics of study participants

		Gender (%)	Age Group (%)				Highest Education Level (%)					
Year			25-34	35-44	45-54	55-64	Degree	A-Level	GCSE	None	Sample	Total Sample
1991	Male	46.6	29.7	27.6	21.9	20.8	16.0	20.1	32.1	31.9	942	
	Female	53.4	30.0	27.2	21.7	21.2	8.1	17.3	34.0	40.6	1078	**2020**
1992	Male	48.4	28.6	28.4	23.8	19.2	15.2	26.0	32.5	26.3	1184	
	Female	51.6	29.8	28.4	21.7	20.1	8.7	18.5	37.9	34.9	1316	**2500**
1993	Male	47.6	29.2	26.1	24.9	19.8	16.4	25.4	30.1	28.2	5030	
	Female	52.4	30.2	27.0	23.8	19.0	9.6	18.2	35.9	36.2	5544	**10,574**
1994	Male	46.7	29.5	27.3	23.0	20.2	15.9	26.5	30.0	27.5	4704	
	Female	53.3	31.0	27.2	23.1	18.8	10.4	18.0	37.9	33.7	5361	**10,065**
1997	Male	47.2	27.7	27.4	25.5	19.4	19.2	28.7	28.3	23.8	2559	
	Female	52.8	30.6	27.2	24.5	17.7	14.0	21.3	33.7	31.0	2860	**5419**
1998	Male	46.6	27.7	26.9	25.8	19.6	19.5	28.0	29.5	23.1	4582	
	Female	53.4	29.0	28.0	24.7	18.3	14.3	20.6	36.0	29.2	5254	**9836**
1999	Male	47.0	25.4	28.1	25.4	21.0	21.0	28.5	27.5	23.0	2257	
	Female	53.0	27.4	29.9	25.7	17.0	15.5	21.6	34.4	28.6	2543	**4800**
2000	Male	45.8	25.9	29.7	23.4	21.0	21.7	30.3	27.0	21.0	2311	
	Female	54.2	27.6	30.5	23.3	18.5	16.5	25.0	34.1	24.4	2733	**5044**
2001	Male	45.6	24.2	28.3	25.6	21.9	22.7	29.2	29.4	18.7	4360	
	Female	54.4	25.6	30.7	25.0	18.7	17.2	23.6	35.3	23.9	5193	**9553**
2002	Male	43.4	23.0	31.5	23.8	21.7	23.1	31.6	28.2	17.2	2016	
	Female	56.6	27.8	32.1	22.0	18.2	19.4	25.1	36.2	19.3	2633	**4649**
2003	Male	45.5	22.6	28.6	24.4	24.3	23.6	28.0	29.7	18.7	4117	
	Female	54.6	23.9	30.6	23.3	22.1	20.4	24.0	34.8	20.8	4941	**9058**

For peer review only

**2004**	Male	43.4	23.2	27.7	22.8	26.3	25.4	27.7	27.1	19.9	1782	
	Female	56.6	21.8	30.3	24.3	23.5	21.1	24.3	31.9	22.8	2327	**4109**
**2005**	Male	44.8	22.0	25.0	26.9	26.0	25.5	29.0	26.7	18.8	2076	
	Female	55.2	22.9	27.9	26.1	23.2	22.1	23.4	32.6	21.9	2555	**4631**
**2006**	Male	44.8	19.9	28.2	25.0	26.9	27.2	29.9	25.6	17.3	3896	
	Female	55.2	22.2	29.2	24.7	23.8	24.2	24.3	32.1	19.4	4795	**8691**
**2008**	Male	44.8	21.1	27.2	24.8	26.9	27.2	30.1	25.9	16.8	4165	
	Female	55.2	22.7	27.9	25.4	24.1	24.5	26.3	30.9	18.3	5135	**9300**
**2009**	Male	45.6	20.7	27.8	25.9	25.7	27.4	26.8	29.3	16.5	1278	
	Female	54.5	22.6	29.7	24.2	23.4	25.9	24.4	32.7	17.2	1528	**2806**
**2010**	Male	43.5	20.3	26.1	26.7	26.8	29.6	30.2	26.6	13.6	2123	
	Female	56.5	22.1	26.8	28.2	22.9	27.5	26.6	32.0	13.9	2759	**4882**
**2012**	Male	43.6	19.8	26.1	26.8	27.4	32.2	29.8	24.3	13.7	2025	
	Female	56.5	22.9	26.1	28.2	22.9	32.3	27.1	26.5	14.1	2625	**4650**
**2014**	Male	43.3	19.2	26.7	29.8	24.3	34.2	27.9	24.6	13.4	1964	
	Female	56.7	22.7	27.6	28.3	21.4	33.6	28.3	25.8	12.4	2568	**4532**

		**Gender (%)**	**Age Group (%)**				**IMD Quintile**						
**Year**			**25-34**	**35-44**	**45-54**	**55-64**	**1 (Least Deprived)**	**2**	**3**	**4**	**5 (Most Deprived)**	**Sample**	**Total Sample**
**2001**	Male	44.8	24.0	27.8	26.1	22.2	16.7	15.2	19.2	21.0	28.0	4211	
	Female	55.2	24.8	29.6	25.6	20.1	16.4	14.8	20.0	22.1	26.8	5184	**9395**
**2002**	Male	42.7	22.9	31.1	23.9	22.1	16.6	16.6	18.6	22.2	26.0	2080	
	Female	57.3	26.8	31.0	22.7	19.6	17.2	16.0	19.0	21.6	26.2	2794	**4874**
**2003**	Male	44.6	22.7	28.4	24.5	24.5	22.6	20.8	19.9	20.7	16.1	4204	
	Female	55.4	23.1	29.7	23.4	23.8	23.2	20.1	19.8	20.7	16.2	5229	**9433**
**2004**	Male	42.9	23.2	27.7	22.8	26.3	23.4	23.2	19.1	20.3	14.1	1786	
	Female	57.1	21.3	29.8	24.3	24.6	22.3	22.8	17.8	19.9	17.2	2380	**4166**

For peer review only - http://bmjopen.bmj.com/site/about/guidelines.xhtml

**2005**	Male	44.3	22.0	25.0	26.9	26.2	23.2	21.3	20.5	20.6	14.5	2082	
	Female	55.7	22.4	27.4	25.9	24.3	22.2	21.6	19.1	20.7	16.4	2618	**4700**
**2006**	Male	44.4	19.8	28.2	25.0	26.9	20.9	21.4	21.4	19.8	16.5	3907	
	Female	55.6	22.0	28.8	24.6	24.7	20.2	22.7	21.0	19.4	16.7	4888	**8795**
**2008**	Male	44.4	21.1	27.2	24.7	27.0	22.3	20.3	19.9	19.4	18.1	4169	
	Female	55.6	22.3	27.5	25.2	25.0	22.6	19.9	20.0	19.2	18.3	5230	**9399**
**2009**	Male	45.1	20.6	27.8	25.8	25.8	20.4	22.4	20.4	20.0	16.9	1282	
	Female	54.9	22.3	29.3	24.3	24.2	20.6	21.4	21.2	20.0	16.7	1559	**2841**
**2010**	Male	43.1	20.3	26.1	26.7	26.9	23.4	19.8	20.0	19.0	17.9	2129	
	Female	56.9	21.9	26.6	28.1	23.6	23.7	19.7	20.0	19.5	17.3	2806	**4935**
**2012**	Male	43.4	19.8	26.1	26.9	27.3	22.1	21.1	20.2	19.3	17.4	2030	
	Female	56.6	22.8	25.9	28.0	23.4	22.7	20.7	19.9	19.2	17.6	2649	**4679**
**2014**	Male	43.2	19.3	26.6	29.9	24.3	23.5	20.3	18.4	18.7	19.1	1969	
	Female	56.8	22.6	27.6	28.1	21.8	21.7	19.6	20.0	19.7	19.1	2592	**4561**

For peer review only

Appendix 2: Age-standardised GHQ caseness in men and women aged 25-64 years, 1991-2014

(displayed in Figure 1 in main text)

	Men				Women			
**Year**	Sample Size	Adjusted Est. (%)	Lower 95% CI	Upper 95% CI	Sample Size	Adjusted Est. (%)	Lower 95% CI	Upper 95% CI
**1991**	942	12.17	10.06	14.28	1078	19.46	17.08	21.84
**1992**	1184	14.48	12.44	16.52	1316	19.71	17.52	21.90
**1993**	5030	14.04	13.07	15.01	5544	18.82	17.78	19.86
**1994**	4704	13.04	12.06	14.02	5361	19.17	18.10	20.24
**1997**	2559	12.77	11.47	14.07	2860	19.06	17.60	20.52
**1998**	4582	13.14	12.15	14.13	5254	17.98	16.94	19.03
**1999**	2257	15.32	13.83	16.81	2543	18.87	17.33	20.41
**2000**	2311	11.97	10.63	13.30	2733	16.44	15.03	17.85
**2001**	4360	11.38	10.43	12.32	5193	15.30	14.31	16.28
**2002**	2016	14.35	12.81	15.89	2633	18.97	17.44	20.50
**2003**	4117	11.86	10.87	12.85	4941	14.80	13.81	15.80
**2004**	1782	11.66	10.16	13.17	2327	15.18	13.71	16.64
**2005**	2076	11.57	10.19	12.94	2555	15.99	14.57	17.41
**2006**	3896	11.95	10.93	12.98	4795	15.58	14.55	16.61
**2008**	4165	11.62	10.64	12.60	5135	16.01	15.01	17.02
**2009**	1278	16.80	14.75	18.86	1528	17.13	15.23	19.04
**2010**	2123	14.58	13.07	16.09	2759	16.66	15.27	18.05
**2012**	2025	12.67	11.22	14.11	2625	18.72	17.22	20.21
**2014**	1964	12.90	11.40	14.40	2568	17.67	16.19	19.15

For peer review only

Appendix 3: Adjusted odds ratio and % point difference in GHQ caseness by gender, 1991-2014

(displayed in Figure 1 in main text)

	Model 1: Adjusted for age			Model 2: Adjusted for age, education, employment		
**Year**	**OR (95% CI)**	**p-value**	**% Diff. (95% CI)**	**OR (95% CI)**	**p-value**	**% Diff. (95% CI)**
**1991**	1.76 (1.38-2.26)	<0.001	7.61 (4.31-10.90)	1.83 (1.39-2.40)	<0.001	7.85 (4.33-11.37)
**1992**	1.48 (1.20-1.83)	<0.001	5.53 (2.53-8.52)	1.57 (1.24-1.98)	<0.001	6.23 (2.95-9.50)
**1993**	1.43 (1.28-1.58)	<0.001	4.91 (3.47-6.35)	1.52 (1.35-1.70)	<0.001	5.64 (4.11-7.17)
**1994**	1.60 (1.44-1.79)	<0.001	6.42 (4.94-7.89)	1.76 (1.57-1.98)	<0.001	7.44 (5.89-9.00)
**1997**	1.60 (1.38-1.85)	<0.001	6.31 (4.31-8.31)	1.68 (1.43-1.98)	<0.001	6.73 (4.63-8.84)
**1998**	1.47 (1.32-1.64)	<0.001	5.11 (3.65-6.58)	1.58 (1.40-1.78)	<0.001	5.79 (4.26-7.33)
**1999**	1.29 (1.11-1.51)	0.001	3.68 (1.52-5.83)	1.41 (1.20-1.67)	<0.001	4.67 (2.42-6.91)
**2000**	1.45 (1.23-1.70)	<0.001	4.56 (2.58-6.54)	1.62 (1.35-1.95)	<0.001	5.52 (3.43-7.61)
**2001**	1.41 (1.25-1.59)	<0.001	4.04 (2.64-5.45)	1.51 (1.32-1.72)	<0.001	4.55 (3.10-6.01)
**2002**	1.41 (1.20-1.65)	<0.001	4.77 (2.55-6.98)	1.50 (1.26-1.79)	<0.001	5.41 (3.11-7.71)
**2003**	1.27 (1.12-1.44)	<0.001	2.74 (1.30-4.17)	1.34 (1.17-1.54)	<0.001	3.19 (1.69-4.69)
**2004**	1.33 (1.10-1.60)	0.004	3.22 (1.07-5.37)	1.36 (1.10-1.69)	0.004	3.32 (1.06-5.57)
**2005**	1.49 (1.25-1.78)	<0.001	4.68 (2.67-6.70)	1.65 (1.36-2.01)	<0.001	5.39 (3.32-7.45)
**2006**	1.32 (1.16-1.50)	<0.001	3.23 (1.73-4.72)	1.39 (1.21-1.60)	<0.001	3.54 (2.03-5.05)
**2008**	1.45 (1.28-1.64)	<0.001	4.35 (2.91-5.79)	1.53 (1.33-1.75)	<0.001	4.53 (3.07-5.99)
**2009**	0.98 (0.80-1.21)	0.866	-0.25 (-3.12-2.62)	1.09 (0.87-1.37)	0.446	1.12 (-1.75-3.98)
**2010**	1.17 (0.99-1.39)	0.065	2.08 (-0.11-4.27)	1.22 (1.01-1.46)	0.034	2.39 (0.19-4.58)
**2012**	1.57 (1.32-1.86)	<0.001	5.93 (3.70-8.15)	1.79 (1.48-2.16)	<0.001	6.78 (4.61-8.94)
**2014**	1.42 (1.19-1.70)	<0.001	4.53 (2.28-6.78)	1.43 (1.19-1.71)	<0.001	4.51 (2.27-6.75)

For peer review only

Appendix 4: Full multiple logistic regression models for participants of each gender, 2001-2014

Regression models for men (n=24,930)												
	Model 1: Adjusted for age, IMD				Model 2: Adjusted for age, IMD, employment				Model 3: Adjusted for age, IMD, employment, income			
**Year**	**Odds Ratio**	**p-value**	**Lower 95% CI**	**Upper 95% CI**	**Odds Ratio**	**p-value**	**Lower 95% CI**	**Upper 95% CI**	**Odds Ratio**	**p-value**	**Lower 95% CI**	**Upper 95% CI**
**2001**	0.93	0.330	0.80	1.08	0.89	0.147	0.76	1.04	0.86	0.069	0.73	1.01
**2002**	1.27	0.007	1.07	1.51	1.29	0.006	1.07	1.54	1.26	0.014	1.05	1.51
**2003**	1.06	0.481	0.91	1.23	0.97	0.667	0.82	1.13	0.94	0.475	0.80	1.11
**2004**	1.11	0.272	0.92	1.35	1.07	0.505	0.87	1.31	1.05	0.646	0.86	1.29
**2005**	1.03	0.722	0.86	1.24	0.92	0.400	0.76	1.12	0.90	0.303	0.74	1.10
**2006**	1.06	0.476	0.91	1.24	1.04	0.676	0.88	1.22	1.03	0.765	0.87	1.21
**2008**	1.00	-	-	-	1.00	-	-	-	1.00	-	-	-
**2009**	1.64	<0.001	1.34	2.00	1.55	<0.001	1.25	1.93	1.53	<0.001	1.24	1.91
**2010**	1.28	0.009	1.06	1.53	1.26	0.021	1.04	1.52	1.26	0.018	1.04	1.53
**2012**	1.15	0.147	0.95	1.38	1.10	0.340	0.91	1.33	1.10	0.342	0.91	1.33
**2014**	1.13	0.215	0.93	1.37	1.17	0.126	0.96	1.43	1.18	0.108	0.96	1.44
**Regression models for women (n=31,413)**												
	Model 1: Adjusted for age, IMD				Model 2: Adjusted for age, IMD, employment				Model 3: Adjusted for age, IMD, employment, income			
**Year**	**Odds Ratio**	**p-value**	**Lower 95% CI**	**Upper 95% CI**	**Odds Ratio**	**p-value**	**Lower 95% CI**	**Upper 95% CI**	**Odds Ratio**	**p-value**	**Lower 95% CI**	**Upper 95% CI**
**2001**	0.94	0.280	0.83	1.05	0.91	0.143	0.81	1.03	0.87	0.030	0.78	0.99
**2002**	1.14	0.049	1.00	1.31	1.13	0.078	0.99	1.30	1.10	0.162	0.96	1.27
**2003**	0.92	0.192	0.82	1.04	0.91	0.112	0.80	1.02	0.87	0.026	0.77	0.98
**2004**	0.93	0.358	0.80	1.08	0.91	0.218	0.78	1.06	0.88	0.112	0.75	1.03
**2005**	1.06	0.426	0.92	1.22	1.05	0.531	0.91	1.21	1.02	0.762	0.88	1.18
**2006**	0.91	0.153	0.81	1.03	0.90	0.102	0.80	1.02	0.89	0.077	0.79	1.01
**2008**	1.00	-	-	-	1.00	-	-	-	1.00	-	-	-
**2009**	1.06	0.537	0.89	1.25	1.07	0.429	0.90	1.28	1.07	0.431	0.90	1.28
**2010**	1.00	0.980	0.86	1.15	0.99	0.849	0.85	1.14	0.99	0.922	0.86	1.15
**2012**	1.24	0.003	1.08	1.42	1.24	0.003	1.07	1.43	1.25	0.002	1.09	1.45
**2014**	1.10	0.208	0.95	1.27	1.11	0.171	0.96	1.28	1.12	0.138	0.97	1.29

For peer review only - http://bmjopen.bmj.com/site/about/guidelines.xhtml

Appendix 5: Age-sex standardised GHQ caseness by IMD quintile in 25–64-year-olds, 2001-2014
(displayed in Figure 2 in main text)

Year	Quintile	Sample Size	Adjusted Est. (%)	Lower 95% CI	Upper 95% CI	Year	Quintile	Sample Size	Adjusted Est. (%)	Lower 95% CI	Upper 95% CI
**2001**	Least Dep.	1549	11.80	10.20	13.41	**2002**	Least Dep.	825	12.65	10.35	14.94
	2	1403	9.36	7.83	10.88		2	794	13.74	11.37	16.11
	3	1846	13.38	11.83	14.94		3	916	13.26	11.11	15.42
	4	2030	14.06	12.56	15.57		4	1065	19.99	17.52	22.46
	Most Dep.	2567	16.42	14.97	17.88		Most Dep.	1274	20.94	18.64	23.24
**2003**	Least Dep.	2163	11.38	10.00	12.75	**2004**	Least Dep.	948	11.98	9.73	14.22
	2	1924	11.19	9.78	12.61		2	957	10.79	8.78	12.80
	3	1872	10.98	9.55	12.40		3	764	11.07	8.87	13.28
	4	1950	16.18	14.54	17.83		4	836	15.66	13.18	18.13
	Most Dep.	1524	18.51	16.53	20.49		Most Dep.	661	19.56	16.43	22.70
**2005**	Least Dep.	1064	9.55	7.73	11.37	**2006**	Least Dep.	1806	10.27	8.81	11.73
	2	1008	12.23	10.22	14.24		2	1943	12.50	11.00	13.99
	3	927	12.18	10.08	14.27		3	1864	11.75	10.28	13.21
	4	970	15.05	12.81	17.28		4	1719	15.71	13.97	17.45
	Most Dep.	731	23.72	20.53	26.92		Most Dep.	1463	20.68	18.56	22.80
**2008**	Least Dep.	2108	10.44	9.10	11.78	**2009**	Least Dep.	582	14.92	11.95	17.90
	2	1889	11.11	9.67	12.54		2	621	11.09	8.62	13.57
	3	1877	12.83	11.33	14.33		3	593	17.91	14.79	21.03
	4	1815	15.12	13.47	16.76		4	568	19.51	16.21	22.81
	Most Dep.	1710	20.54	18.59	22.50		Most Dep.	477	24.07	20.06	28.08
**2010**	Least Dep.	1162	13.73	11.66	15.81	**2012**	Least Dep.	1048	10.67	8.72	12.62
	2	973	11.60	9.55	13.65		2	977	14.40	12.22	16.58
	3	985	15.53	13.28	17.78		3	937	14.50	12.26	16.75
	4	951	17.48	15.05	19.92		4	899	16.83	14.40	19.26
	Most Dep.	864	22.28	19.47	25.09		Most Dep.	818	24.18	21.21	27.16
**2014**	Least Dep.	1024	10.71	8.80	12.62						
	2	907	13.52	11.27	15.76						
	3	880	15.30	12.93	17.67						
	4	879	16.04	13.61	18.48						
	Most Dep.	871	21.91	19.10	24.73						

For peer review only

Appendix 6: Age-sex standardised GHQ caseness by education level in 25–64-year-olds, 1991-2014

(displayed in Figure 3 in main text)

Year	Educ. Level	Sample Size	Adjusted Est. (%)	Lower 95% CI	Upper 95% CI	Year	Educ. Level	Sample Size	Adjusted Est. (%)	Lower 95% CI	Upper 95% CI
**1991**	Degree	238	16.92	11.61	22.24	**1992**	Degree	295	16.88	12.67	21.09
	A-Level	375	19.29	14.49	24.08		A-Level	551	16.69	13.28	20.10
	GCSE	669	13.85	11.15	16.54		GCSE	884	17.08	14.36	19.79
	None	738	15.65	12.81	18.48		None	770	16.57	13.83	19.30
**1993**	Degree	1356	18.18	15.56	20.79	**1994**	Degree	1308	17.61	15.39	19.82
	A-Level	2286	15.55	13.96	17.14		A-Level	2213	15.20	13.57	16.83
	GCSE	3508	15.90	14.61	17.18		GCSE	3445	14.83	13.55	16.11
	None	3424	17.34	15.98	18.70		None	3099	17.03	15.65	18.41
**1997**	Degree	892	12.23	9.97	14.49	**1998**	Degree	1642	14.66	12.75	16.56
	A-Level	1344	16.23	14.13	18.32		A-Level	2362	15.38	13.88	16.88
	GCSE	1687	15.01	13.21	16.80		GCSE	3243	15.23	13.90	16.56
	None	1496	16.69	14.69	18.70		None	2589	16.92	15.30	18.53
**1999**	Degree	868	15.62	13.07	18.17	**2000**	Degree	952	12.50	10.31	14.68
	A-Level	1193	15.32	13.20	17.45		A-Level	1382	10.75	9.08	12.42
	GCSE	1494	16.36	14.37	18.36		GCSE	1558	14.38	12.53	16.23
	None	1245	21.39	18.80	23.98		None	1152	19.12	16.45	21.80
**2001**	Degree	1881	12.40	10.82	13.99	**2002**	Degree	975	14.01	11.80	16.23
	A-Level	2501	12.87	11.54	14.20		A-Level	1299	15.52	13.45	17.59
	GCSE	3115	12.47	11.27	13.67		GCSE	1520	16.46	14.46	18.46
	None	2056	15.93	14.08	17.78		None	855	22.12	18.83	25.42
**2003**	Degree	1982	11.25	9.81	12.68	**2004**	Degree	943	13.10	10.86	15.35
	A-Level	2339	12.22	10.87	13.57		A-Level	1058	12.41	10.44	14.39
	GCSE	2940	12.98	11.74	14.22		GCSE	1224	10.94	9.15	12.73
	None	1797	17.16	15.05	19.27		None	884	18.26	15.36	21.17
**2005**	Degree	1094	10.98	9.08	12.87	**2006**	Degree	2217	10.51	9.19	11.83
	A-Level	1199	12.65	10.76	14.53		A-Level	2332	13.37	11.98	14.76
	GCSE	1389	13.56	11.75	15.37		GCSE	2536	13.73	12.36	15.11
	None	949	19.51	16.52	22.50		None	1606	19.69	17.47	21.91
**2008**	Degree	2388	11.04	9.76	12.32	**2009**	Degree	745	13.01	10.43	15.59
	A-Level	2604	12.55	11.28	13.83		A-Level	715	16.15	13.42	18.87
	GCSE	2669	14.82	13.44	16.20		GCSE	873	18.41	15.76	21.06
	None	1639	19.43	17.18	21.68		None	473	21.60	17.46	25.74
**2010**	Degree	1387	13.21	11.40	15.02	**2012**	Degree	1500	14.63	12.85	16.41
	A-Level	1375	15.43	13.51	17.35		A-Level	1314	14.17	12.30	16.05
	GCSE	1447	15.56	13.64	17.49		GCSE	1188	16.87	14.69	19.06
	None	673	21.54	17.86	25.22		None	648	19.35	16.07	22.63
**2014**	Degree	1533	12.96	11.25	14.67						
	A-Level	1273	13.61	11.68	15.53						

	GCSE	1145	15.13	13.00	17.26						
	None	581	23.71	20.04	27.38						

For peer review only

Appendix 7: Relative index of inequality in GHQ caseness, 1991-2014

(displayed in Figure 4 in main text)

Highest Education Level as Measure of SEP					Area-Level Deprivation as Measure of SEP				
Year	Incidence Rate Ratio	p-value	Lower 95% CI	Upper 95% CI	Year	Incidence Rate Ratio	p-value	Lower 95% CI	Upper 95% CI
**1991**	0.86	0.445	0.58	1.27					
**1992**	0.88	0.458	0.63	1.23					
**1993**	1.02	0.797	0.87	1.21					
**1994**	0.96	0.634	0.81	1.14					
**1997**	1.25	0.054	1.00	1.57					
**1998**	1.11	0.232	0.93	1.32					
**1999**	1.46	0.002	1.15	1.86					
**2000**	1.79	<0.001	1.37	2.33					
**2001**	1.26	0.018	1.04	1.53	**2001**	1.68	<0.001	1.40	2.02
**2002**	1.51	0.001	1.18	1.93	**2002**	1.93	<0.001	1.54	2.41
**2003**	1.57	<0.001	1.27	1.92	**2003**	1.95	<0.001	1.61	2.37
**2004**	1.35	0.066	0.98	1.86	**2004**	2.05	<0.001	1.52	2.77
**2005**	1.70	<0.001	1.29	2.24	**2005**	2.65	<0.001	2.03	3.47
**2006**	1.98	<0.001	1.61	2.43	**2006**	2.13	<0.001	1.74	2.60
**2008**	1.79	<0.001	1.47	2.18	**2008**	2.28	<0.001	1.89	2.76
**2009**	2.13	<0.001	1.52	2.99	**2009**	2.11	<0.001	1.54	2.91
**2010**	1.59	0.001	1.22	2.08	**2010**	1.85	<0.001	1.43	2.38
**2012**	1.46	0.005	1.12	1.90	**2012**	2.32	<0.001	1.80	2.99
**2014**	1.72	<0.001	1.31	2.26	**2014**	2.22	<0.001	1.72	2.87

For peer review only

STROBE Statement—Checklist of items that should be included in reports of ***cross-sectional studies***

	Item No	Recommendation	Page/ Line
**Title and abstract**	1	(*a*) Indicate the study's design with a commonly used term in the title or the abstract	p1, ln1-3
		(*b*) Provide in the abstract an informative and balanced summary of what was done and what was found	p2, ln2-25
**Introduction**			
Background/rationale	2	Explain the scientific background and rationale for the investigation being reported	p3, ln2-27
Objectives	3	State specific objectives, including any prespecified hypotheses	p3, ln28-30
**Methods**			
Study design	4	Present key elements of study design early in the paper	p4, ln3-5
Setting	5	Describe the setting, locations, and relevant dates, including periods of recruitment, exposure, follow-up, and data collection	p4, ln3-7
Participants	6	(*a*) Give the eligibility criteria, and the sources and methods of selection of participants	p4, ln11-15
Variables	7	Clearly define all outcomes, exposures, predictors, potential confounders, and effect modifiers. Give diagnostic criteria, if applicable	p4, ln24-30<br>p5, ln1-15
Data sources/ measurement	8*	For each variable of interest, give sources of data and details of methods of assessment (measurement). Describe comparability of assessment methods if there is more than one group	p5, ln24-30<br>p6, ln1-15
Bias	9	Describe any efforts to address potential sources of bias	p5, ln7<br>p5, ln14-15<br>p6, ln10-11
Study size	10	Explain how the study size was arrived at	n/a
Quantitative variables	11	Explain how quantitative variables were handled in the analyses. If applicable, describe which groupings were chosen and why	p6, ln2
Statistical methods	12	(*a*) Describe all statistical methods, including those used to control for confounding	p6, ln17-32<br>p7, ln1-7
		(*b*) Describe any methods used to examine subgroups and interactions	p6, ln17-32<br>p7, ln1-7
		(*c*) Explain how missing data were addressed	p5, ln12-14
		(*d*) If applicable, describe analytical methods taking account of sampling strategy	p5, ln7
		(*e*) Describe any sensitivity analyses	p5, ln14-15
**Results**			
Participants	13*	(a) Report numbers of individuals at each stage of study—eg numbers potentially eligible, examined for eligibility, confirmed eligible, included in the study, completing follow-up, and analysed	p5, ln16-22
		(b) Give reasons for non-participation at each stage	p5, ln16-22
		(c) Consider use of a flow diagram	n/a
Descriptive data	14*	(a) Give characteristics of study participants (eg demographic, clinical, social) and information on exposures and potential confounders	p8, ln2-5
		(b) Indicate number of participants with missing data for each variable of interest	p5, ln16-22
Outcome data	15*	Report numbers of outcome events or summary measures	p8-p11

For peer review only

Main results	16	(*a*) Give unadjusted estimates and, if applicable, confounder-adjusted estimates and their precision (eg, 95% confidence interval). Make clear which confounders were adjusted for and why they were included	Table 1
		(*b*) Report category boundaries when continuous variables were categorized	p6, ln2
		(*c*) If relevant, consider translating estimates of relative risk into absolute risk for a meaningful time period	Table 1
Other analyses	17	Report other analyses done—eg analyses of subgroups and interactions, and sensitivity analyses	p10, ln25-30 p11, ln10
**Discussion**			
Key results	18	Summarise key results with reference to study objectives	p12, ln2-7
Limitations	19	Discuss limitations of the study, taking into account sources of potential bias or imprecision. Discuss both direction and magnitude of any potential bias	p13, ln24-33 p14, ln1-3
Interpretation	20	Give a cautious overall interpretation of results considering objectives, limitations, multiplicity of analyses, results from similar studies, and other relevant evidence	Throughout
Generalisability	21	Discuss the generalisability (external validity) of the study results	p14, ln26-28
**Other information**			
Funding	22	Give the source of funding and the role of the funders for the present study and, if applicable, for the original study on which the present article is based	p15, ln14-16

*Give information separately for exposed and unexposed groups.

Note: An Explanation and Elaboration article discusses each checklist item and gives methodological background and published examples of transparent reporting. The STROBE checklist is best used in conjunction with this article (freely available on the Web sites of PLoS Medicine at http://www.plosmedicine.org/, Annals of Internal Medicine at http://www.annals.org/, and Epidemiology at http://www.epidem.com/). Information on the STROBE Initiative is available at www.strobe-statement.org.

For peer review only
